# Intestinal Neutral Ceramidase Deficiency Triggers Regulatory T Cell Response via Gd3 to Protect the Host from Intestinal Inflammation

**DOI:** 10.1002/advs.202512681

**Published:** 2025-11-07

**Authors:** Zhishan Xu, Chao Lei, Mukesh K Sriwastva, Ting Wang, Amanguli Tuohongerbieke, Xiaotong Song, Collin Noud, Shannon Derkson, Yi Tan, Gerald Dryden, Craig J McClain, Zhongbin Deng

**Affiliations:** ^1^ Department of Surgery Division of Immunotherapy University of Louisville Louisville KY 40202 USA; ^2^ Brown Cancer Center University of Louisville Louisville KY 40202 USA; ^3^ Department of Immunology School of Basic Medical Sciences Xinxiang Medical University Xinxiang 453003 China; ^4^ Department of Medicine University of Louisville Louisville KY 40202 USA; ^5^ Pediatric Research Institute Department of Pediatrics University of Louisville Louisville KY 40202 USA; ^6^ Alcohol Research Center University of Louisville Louisville KY 40202 USA; ^7^ Hepatobiology & Toxicology Center University of Louisville Louisville KY 40202 USA; ^8^ Robley Rex VA Medical Center Louisville KY 40202 USA

**Keywords:** colitis, gangliosides, macrophages, Siglecs, ST8SIA1

## Abstract

Sphingolipids play a crucial role in gut inflammation. Neutral ceramidase (NcDase) serves as a pivotal regulator of ceramide, the central intermediate in sphingolipid metabolism. The contribution of intestinal epithelial cells (IEC) NcDase to colitis is not well understood. Here, a protective mechanism by which IEC NcDase deficiency (*Asah2* ^ΔIEC^) and its‐related gangliosides prevent dextran sulfate sodium (DSS)‐induced colitis in mice is described. *Asah2* ^ΔIEC^ mice display reduced susceptibility to DSS‐induced colitis and increase regulatory T (T_reg_) cells compared to *Asah2* ^fl/fl^ littermates. Deletion of IEC NcDase induces the upregulation of sialyltransferase ST8SIA1 and promotes the sialic‐acid‐containing ganglioside GD3 production. Siglec‐E is a sialic‐acid‐binding lectin expresses predominantly on myeloid cells. Mechanistically, it is identified that GD3 is a functional ligand for Siglec‐E on macrophages and found that GD3/Siglec‐E interaction induced a rapid metabolic rewiring of macrophages that involved the production of IL‐33, which contributes to the generation of ST2^+^Foxp3^+^ T_reg_ cells. Finally, deletion of ST8SIA1 or administration of dietary GD3 induces or reduces mucosal inflammation, respectively. This work defines a critical role for ganglioside GD3 in the induction of colonic T_reg_ cells and identifies an activating pathway that follows engagement of Siglec‐E.

## Graphical abstract

Deletion of ASAH2 in intestinal epithelial cells induces the accumulation of ganglioside GD3 via regulating ST8SIA1. GD3 is identified as the glycolipid ligand for the inhibitory receptor Siglec‐E on macrophages. GD3/Siglec‐E ligation polarizes macrophages and promotes the proliferation of colonic ST2^+^ T regulatory cells via the induction of IL‐33. The administration of dietary GD3 reduces mucosal inflammation via ST2^+^ Treg cells.

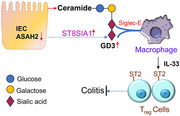

## 1 Introduction

Complex sphingolipids, such as ceramide and its‐related glycosphingolipids (GSLs), are essential components of intestinal membranes and provide protection and integrity to the mucosa. Neutral ceramidase (NcDase), encoded by *Asah2*, is particularly highly expressed in the gut and liver and responsible for ceramide degradation.^[^ ^1^ ^]^ We and others have previously shown that global loss of neutral ceramidase promotes gut inflammation in mouse models of inflammatory bowel disease (IBD)^[^ ^2^ ^]^ and enteric infection.^[^ ^3^ ^]^ The potential roles of NcDase in intestinal epithelial cells (IECs) in the pathogenesis of IBD, however, have not been studied yet.

Epithelial glycans contribute to intestinal mucosal homeostasis.^[^ ^4^, ^5^ ^]^ Glycosphingolipids are major constituents of enterocytes and participate in epithelial barrier integrity.^[^ ^6^, ^7^ ^]^ Glycosylation of ceramide by UDP‐glucose ceramide glucosyltransferase (*Ugcg*) results in glucosylceramide (GlcCer), which is further transformed into lactosylceramide (LacCer).^[^ ^8^ ^]^ Gangliosides are synthesized by the stepwise addition of carbohydrates to LacCer, including one or several sialic acids by several sialyltransferases.^[^ ^9^ ^]^ Sialyltransferase ST8SIA1 catalyzes the transfer of sialic acid from CMP‐sialic acid to ganglioside GM3 to produce GD3. Glycosylation pattern is associated with colitis development.^[^ ^5^ ^]^ For example, decreases in sialic acid‐containing proteins and lipids intensify with IBD progression and enhance gut inflammation in the context of an intact immune system in mice.^[^ ^10^, ^11^ ^]^ GD3 species have been found depleted in the intestine in patients with ulcerative colitis or inflammatory Crohn's disease.^[^ ^12^, ^13^ ^]^ Although dietary gangliosides have been reported to inhibit the degradation of occludin and decrease intestinal permeability in preclinical models of IBD,^[^ ^14^, ^15^, ^16^ ^]^ the mechanisms underlying the mitigation of pro‐inflammatory signaling by dietary gangliosides remain largely unknown. In addition, how these endogenous gangliosides at epithelial surfaces play physiological functions on IBD is largely unexplored.

Siglecs (‘sialic‐acid‐binding immunoglobulin‐like lectins’) are a family of sialic‐acid‐binding lectins that are expressed on various cells of the immune system.^[^ ^17^ ^]^ The cytoplasmic domains of most Siglecs contain immunoreceptor tyrosine‐based inhibitory motifs and thus regulate the cells of the innate and adaptive immune responses.^[^ ^18^ ^]^ Siglec‐9 and murine Siglec‐E, the murine functional homolog of Siglec‐9, have a ‘preference’ for sialic acid α2,3 linked to galactose on myeloid cells.^[^ ^19^ ^]^ The expression of inhibitory human Siglec‐9 and murine Siglec‐E is shown to drive macrophage polarization toward the M2 phenotype. Lamina propria (LP) derived macrophages (LP‐macrophage) have been shown to be less prone to the development of inflammatory responses.^[^ ^20^ ^]^ However, little is known about the induction of tolerogenic effects through the sialic acid‐containing glycosphingolipid‐Siglecs axis on intestinal mucosal immunity. LP‐macrophages and LP‐DCs can actively participate in the de novo generation of CD4^+^CD25^+^Foxp3^+^ regulatory T (T_reg_) cells in the gut.^[^ ^20^, ^21^ ^]^ T_reg_ cells serve a central role in anti‐inflammatory responses in the gut. How the T_reg_ cell population at barrier surfaces is controlled is incompletely understood. The local gut microenvironment plays a major role in controlling the development of inflammatory reactions and the induction of tolerance. Here, we found that during the interaction of macrophages with IECs, IEC‐derived gangliosides could participate in driving the tolerogenic mucosal phenotype of gut macrophages via Siglecs. Specifically, our study showed that epithelial NcDase‐related metabolite GD3, a sialic acid‐containing glycosphingolipid, is a functional ligand for Siglec‐E on macrophages. GD3/Siglec‐E interaction facilitates the proliferation of T_reg_ cells through the engagement of IL‐33 produced by macrophages and inhibits the development of colitis.

## 2 Results

### 2.1 NcDase Deletion in IECs Attenuates DSS‐Induced Colitis

**Figure 1:**
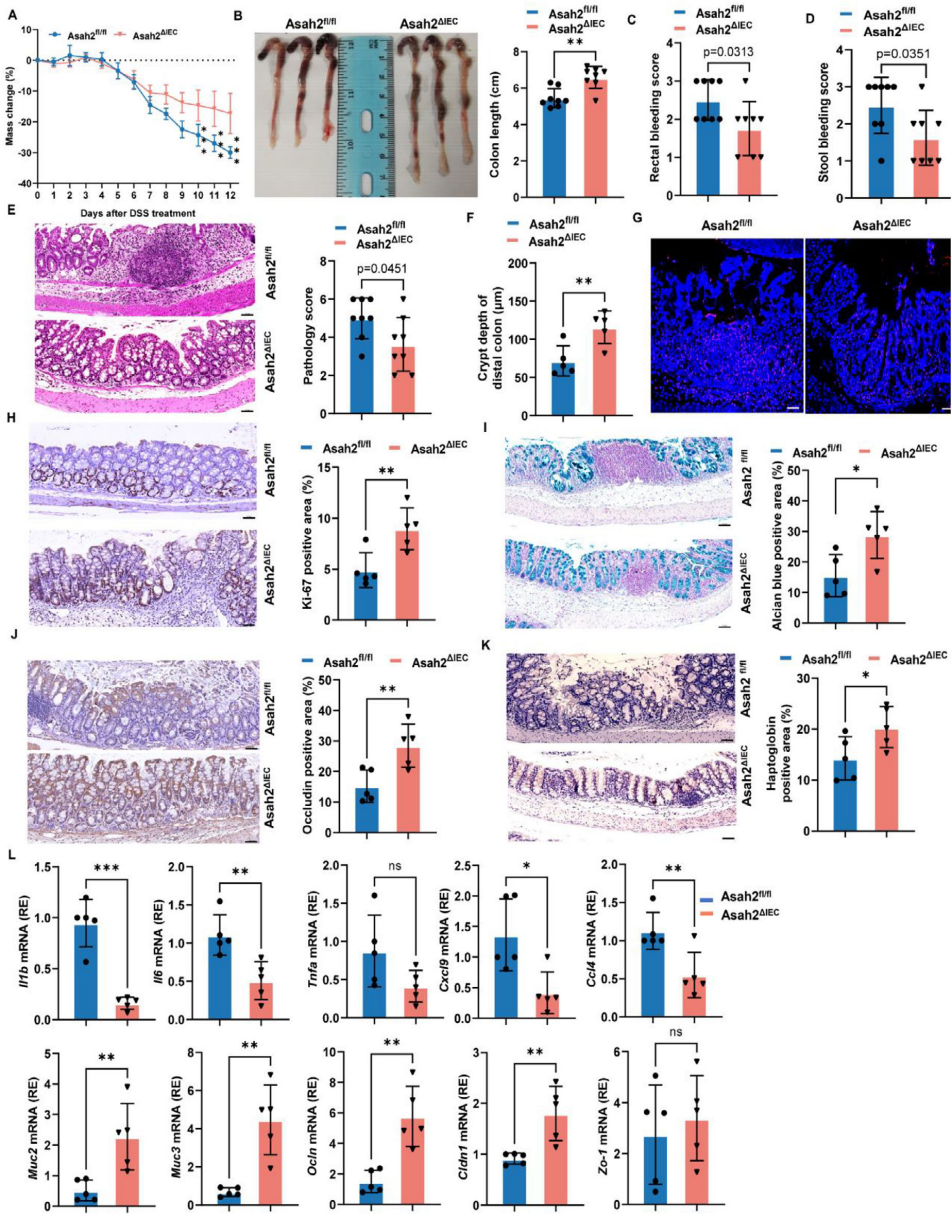
Lack of IEC neutral ceramidase prevents DSS‐induced colitis. *Asah2^ΔIEC^* and *Asah2^fl/fl^* mice were treated with 2.5% DSS in drinking water for 12 days. A) Weight loss in animals following the induction of colitis, measured as a reduction from initial weight until the day of sacrifice. Data were analyzed by two‐way repeated‐measures ANOVA followed by Tukey's posttest. B) Colon images and length. C,D) Scores for rectal bleeding (C) and blood in stools (D). E) Representative histology staining and histological score of colons. F) Crypt depth of the distal colon. G–I) TUNEL (G) and Ki67 (H), and alcian blue (I) staining of the colon. J,K) Immunohistochemistry staining of occludin (J) and haptoglobin (K) proteins in the colon. L) Real‐time PCR analysis of indicated genes in the colon. Statistical comparisons were performed using a two‐tailed unpaired *t*‐test, Error bars indicate mean ± SD. *p < 0.05, **p < 0.01, ***p < 0.001. *n *= 8 (A). Each dot represents one mouse (B–D). Scale bar represents 50 µm.

To investigate the influence of IEC NcDase deficiency on mucosal inflammation, we generated *Asah2^ΔIEC^* mice expressing Villin‐Cre and *Asah2‐*lox alleles (*Asah2^fl/fl^*) and used a DSS‐induced colitis model. *Asah2^ΔIEC^* mice showed significantly decreased weight loss, colon shortening, rectal bleeding, and blood score in the stools (**Figure** **1**A–D; Figure S1A‐‐C, Supporting Information) compared to *Asah2^fl/fl^* mice after the administration of 2.5% DSS for 12 days. Histological examination showed *Asah2^ΔIEC^* mice exhibited reduced cellular infiltration into the mucosa with less mucosal erosion and resultant crypt loss in the large intestine compared to the *Asah2^fl/fl^* mice (Figure 1E,F). We also analyzed cell proliferation and apoptosis by TUNEL and Ki67 staining, respectively. *Asah2^ΔIEC^* mice showed a higher epithelial cell proliferation with noticeable impact on cell apoptosis in the DSS‐induced colitis model (Figure 1G,H). The number of alcian blue^+^ goblet cells and Occludin^+^ and Haptoglobin^+^ IECs was significantly increased (Figure 1I–K). These data suggest that deletion of NcDase induces epithelial cell proliferation to promote the repair of damaged intestinal epithelium and decreases intestinal permeability. The mRNA expression of inflammatory cytokines and chemokines (e.g., Il1b, Il6, Tnfa, Cxcl9, and Ccl4) was also decreased in the colons of *Asah2^ΔIEC^* mice. However, *Asah2^ΔIEC^* colon showed significantly increased expression of the genes encoding mucins and tight junction proteins compared to control mice (Figure 1L). These results indicate that deletion of NcDase in IECs prevents the development of DSS‐induced colitis.

### 2.2 Epithelial NcDase Deficiency is Associated with Increases in the Number of Colonic Treg Cells and the Production of GD3

**Figure 2:**
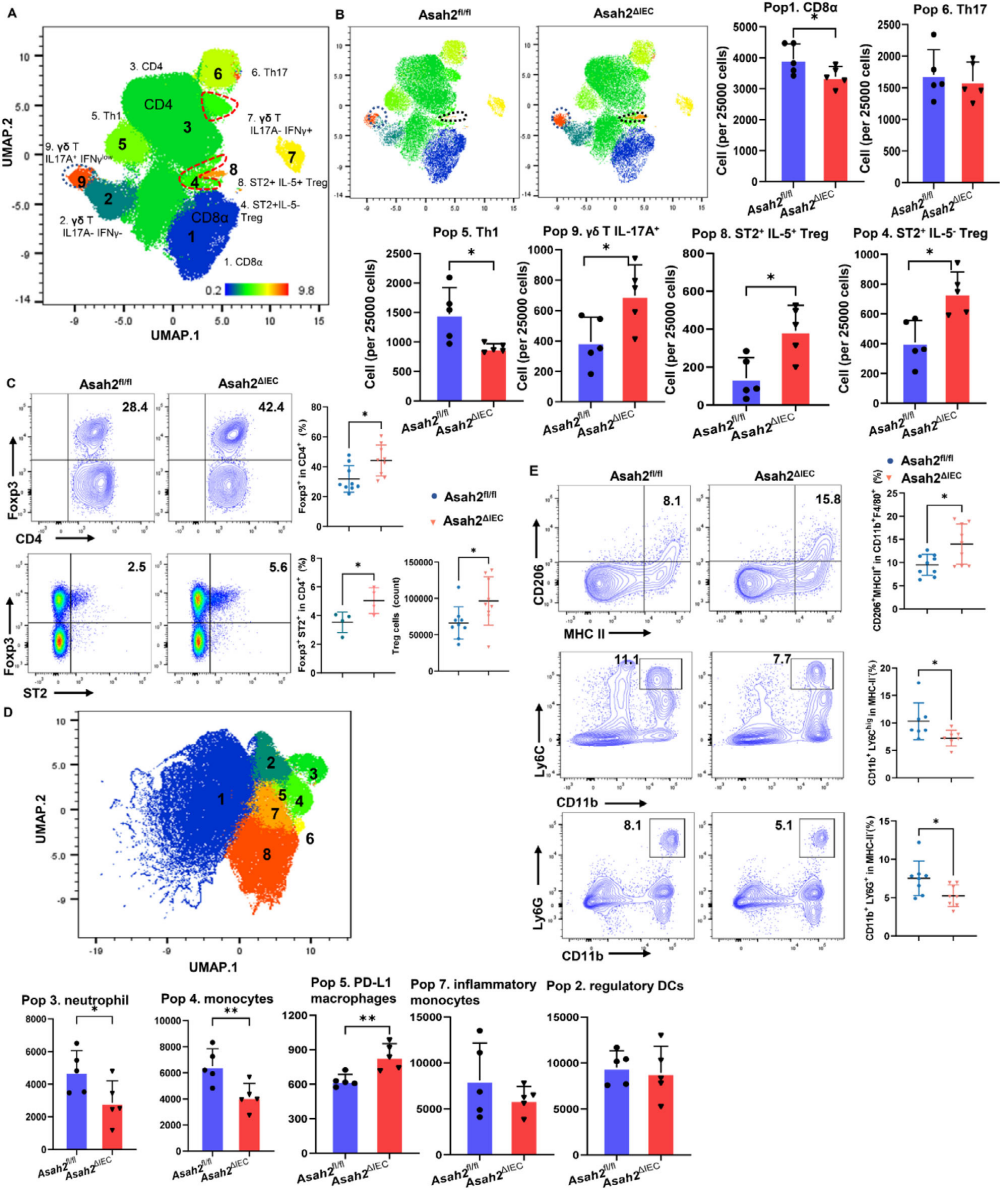
Lack of IEC neutral ceramidase increases the ST2^+^Foxp3^+^ T_reg_ cell population in the colon. A) Schematic representation of clustering after CyTOF analysis of lamina propria lymphocytes (LPL) from large intestine (LI‐LPL) in DSS‐treated *Asah2^ΔIEC^* and *Asah2^fl/fl^* mice. B) Dot plot representation of UMAP analysis and analysis of 25,000 cells showing different clusters that enable the distinction of CD45^+^ CD3^+^ T cell populations in LI‐LPL. Images made with CyTOF data. *n* = 5 independent biological samples. C) Flow cytometry analysis of the percentage and number of Foxp3^+^ T_reg_ and ST2^+^Foxp3^+^ T_reg_ cells in the colon. D) Dot plot representation of UMAP analysis and analysis of 98 000 cells showing different clusters that enable the distinction of CD45^+^ CD11b^+^ myeloid cell populations in LI‐LPL. E) Flow cytometry analysis of myeloid cell populations in the colon. Statistical comparisons were performed using a two‐tailed unpaired *t‐*test. Error bars indicate mean ± SD. *p < 0.05, **p < 0.01. Each dot represents one mouse (C,E).

To explore which immune cell population was mainly affected by the epithelial NcDase deficiency, we first performed cytometry by time of flight (CyTOF) of CD3‐enriched T cells in the distal colonic lamina propria of *Asah2^fl/fl^* and *Asah2^ΔIEC^* littermate controls and identified nine clusters by uniform manifold approximation and projection (UMAP) (**Figure** **2**A; Figure S1D, Supporting Information). The expression patterns of populations 5 and 6 correlated with Th1 cells and Th17 cells, respectively. *Asah2^ΔIEC^* mice have a decrease in Th1 cells and similar Th17 cells and Th2 cells in the colon compared to controls (Figure 2B; Figure S1E, Supporting Information). γδT cells were divided into three clusters noted as population 2 (gdT IL17A^−^IFNγ^−^), population 7 (gdT IL17A^−^IFNγ^+^), and population 9 (gdT IL17A^+^IFNγ^low^). *Asah2^ΔIEC^* mice had a higher number of gdT IL17A^+^ IFNγ^low^ cells (population 9). Population 4 expressed both Foxp3 and ST2 but not IL‐5, corresponding to T_reg_ cells. The number of T_reg_ cells (populations 4 and 8), particularly population 8 ST2^+^ IL‐5^+^ Foxp3^+^ T_reg_ cells, was significantly increased in *Asah2^ΔIEC^* mice (Figure 2B). Indeed, flow cytometry confirmed that *Asah2^ΔIEC^* mice were characterized by higher T_reg_ cells, particularly ST2^+^ Foxp3^+^T_reg_ cells, but not helios^+^ Foxp3^+^T_reg_ cells in the colon lamina propria compared to controls (Figure 2C; Figure S1F, Supporting Information). We also assessed the myeloid cells by CyTOF and flow cytometry and analyzed by UMAP and flowSOM (Figure 2D; Figure S1G, Supporting Information). The expression patterns of populations 3 and 4 correlated with neutrophils (CD11b^+^Ly6G^+^Ly6C^int^) and monocytes (CD11b^+^Ly6C^hi^Ly6G^−^F4/80^int^MHCII^int^), respectively (Figure 2D). The CyTOF data (Figure 2D) and flow cytometry (Figure 2E) showed there were significantly fewer neutrophils and monocytes in the colon of *Asah2^ΔIEC^* mice. However, the PD‐L1^+^/CD206^+^ macrophages (population 5) were increased in *Asah2^ΔIEC^* mice (Figure 2D,E). *Asah2^ΔIEC^* mice have similar frequency in Population 2 DCs expressing CD11b^+^, CD11c^+^, MHCII^hi^, F4/80^−^CD103^low^ and PD‐L1^hi^ compared to *Asah2^fl/fl^* mice (Figure 2D; Figure S1H, Supporting Information).

To further determine whether IEC NcDase‐related macrophages are the effector cells that control gut T_reg_ cell production and intestinal inflammation, we depleted macrophages with clodronate liposome (Clodrosome) in *Asah2^ΔIEC^* and *Asah2^fl/fl^* mice during the induction of colitis. Depletion of macrophages led to a shorter colon length and more gut inflammation *Asah2^ΔIEC^* mice, which is similar to that seen in *Asah2^fl/fl^* mice (Figure S2A,B, Supporting Information). These data are consistent with the comparable levels of T_reg_ cells in the colon between *Asah2^fl/fl^* mice and *Asah2^ΔIEC^* mice (Figure S2C, Supporting Information). Together, these results support a requirement for antigen‐presenting cells (APCs) in the induction of colonic T_reg_ cells and suggest a T cell‐extrinsic immunoregulatory mechanism.

**Figure 3:**
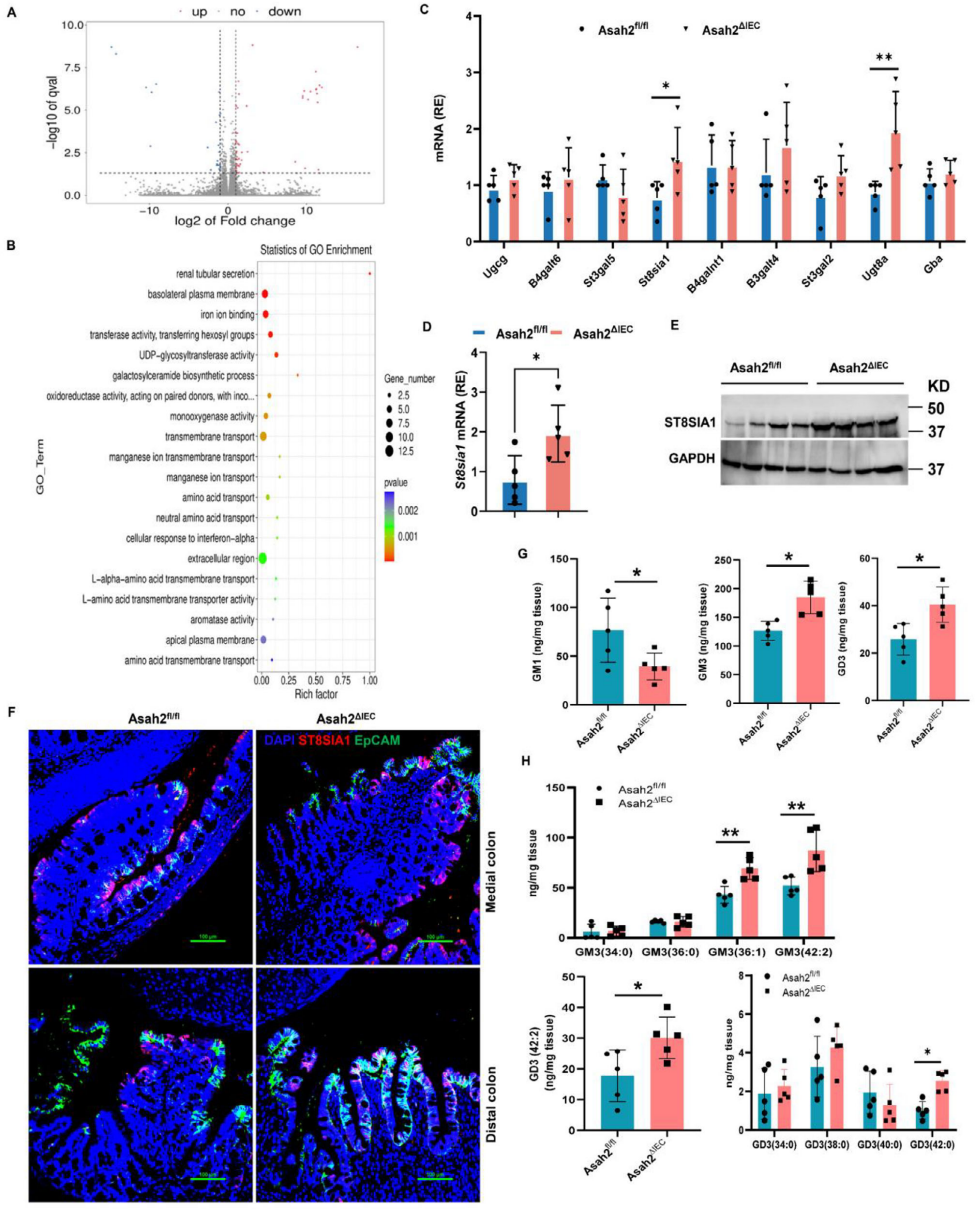
Deletion of IEC neutral ceramidase increases the expression of *St8sia1* and promotes the production of GD3 in the colon. A) Volcano plots showing fold change (FC) and p‐value for the comparison of in IECs isolated from the colon of DSS‐treated *Asah2^ΔIEC^* and *Asah2^fl/fl^* mice. Genes up‐ or downregulated in IECs (at FC > 2 and *p* < 0.05) are highlighted (red and blue). B) GO enrichment analysis of genes defining each pathway is represented in the accompanying bubble plot. Bubble colors represent the corrected *p*‐values. Bubble sizes indicate the number of genes. C) Real‐time PCR analysis of the genes involved in glycosphingolipid and ganglioside synthesis pathway in the IECs of the colon. D–F) Real‐time PCR (D), western blot (E), and immunofluorescence (F) analysis of the St8sia1 expression in the ileum (D) or colon (E,F). G,H) Levels of gangliosides in the colon from DSS‐treated *Asah2^ΔIEC^* and *Asah2^fl/fl^* mice. Statistical comparisons were performed using a two‐tailed unpaired *t‐*test. Error bars indicate mean ± SD. *p < 0.05, **p < 0.01. *n* = 3 (A,B), *n* = 4 (E), and *n* = 5 (C,D,G,H) independent biological samples. Scale bar represents 100 µm.

We next explored potential mechanisms by which the IECs' NcDase may be linked to the interaction between colonic macrophages and T_reg_ cells and the associated intestinal inflammation. To this aim, we performed bulk RNA sequencing (RNA‐seq) of IEC samples from the distal colon of *Asah2^fl/fl^* and *Asah2^ΔIE^* ^C^ littermates, on day 6 after the induction of DSS colitis. The transcriptomic landscape of *Asah2^ΔIEC^* mice was different from that of *Asah2^fl/fl^* mice after DSS supplementation (**Figure** **3**A,B). GO (Gene Ontology) analysis of differentially expressed genes identified pathways related to glycosphingolipid metabolism (*Ugt8a, Ugt3a2, Ugt1a8, B3galt2, B3galt5*) (Figure 3A,B; Figure S3A, Supporting Information). Multiple significantly changed genes in *Asah2^ΔIEC^* mice were noted in comparison to *Asah2^fl/fl^* littermate controls, including *Slc30a10, Slc38a1, Retnlb, Aqp4*, *and Nxpe4*, which are involved in amino acid transport and manganese ion transport (Figure S3A, Supporting Information). Inactivation of NcDase also increased the levels of iron ion binding and transport‐related genes (*Slc11a2, Cyp2d26, Cyp3a44, scn2b*, and *Best2)*, and the basolateral plasma membrane‐related genes (*Slc38a1, Slc9a4, Slc51a, Aqp4*, and *S100g*) (Figure S3A, Supporting Information). Next, we compared the expression of members of the glycosphingolipids/ganglioside biosynthetic pathway by real‐time PCR. The expression of *Ugt8a*, the galactosyltransferase responsible for the synthesis of galactosylceramide (GalCer), and the expression of *St8sia1*, the sialyltransferase that catalyzes the transfer of sialic acid from CMP‐sialic acid to ganglioside GM3 to produce ganglioside GD3, increased in the colonic IECs and ileum of *Asah2^ΔIEC^* mice (Figure 3C,D; Figure S3B, Supporting Information). However, the level of *Ugcg* mRNA, which encodes an enzyme that catalyzes the first glycosylation step in the biosynthesis of glycosphingolipids, was expressed similarly (Figure 3C; Figure S3B, Supporting Information). At the later overt inflammatory phase (day 10 after the start of DSS supplementation), *Asah2^ΔIEC^* mice featured significantly increased expression of St8sia1 in distal colon tissue homogenates compared to littermate controls, as evidenced by western blotting (Figure 3E; Figure S3C, Supporting Information). We also stained epithelial tissues from the colon with an antibody to St8sia1, and anti‐EpCAM, and detected higher levels of St8sia1^+^ in the colonic epithelium of *Asah2^ΔIEC^* mice than that of *Asah2^fl/fl^* (Figure 3F). Next, we performed quantitative lipidomic analysis of gangliosides in the colon using HPLC coupled to a Thermo LTQ Orbitrap XL mass spectrometer and determined the levels of gangliosides in the gut of the *Asah2^ΔIEC^* and *Asah2^fl/fl^* mice normalized to a GM1 standard. Higher amounts of total GM3 and GD3 gangliosides were detected in the gut of the *Asah2^ΔIEC^* mice compared to *Asah2^fl/fl^* mice (Figure 3G). The GM3 36:1, GM3 42:2, GD3 42:0, and GD3 42:2 are the most abundant species with all of them significantly increased in the colon of *Asah2^ΔIEC^* mice (Figure 3H). However, the total amount of GM1 was much lower in the colon of *Asah2^ΔIEC^* mice (Figure 3G). G1a/b, GT1a/b, and GQ1b showed similar amount in *Asah2^ΔIEC^* and *Asah2^fl/fl^* mice (Figure S3D, Supporting Information). The GD2, GT2, and GT3, were not detected in the gut (data not shown). Taken together, these data indicate that IEC‐derived glycosphingolipids and their related gangliosides might contribute to intestinal inflammation and mucosal damage.

### 2.3 GD3 is a Lipid Ligand for Siglec‐E Expressed by Macrophages

**Figure 4:**
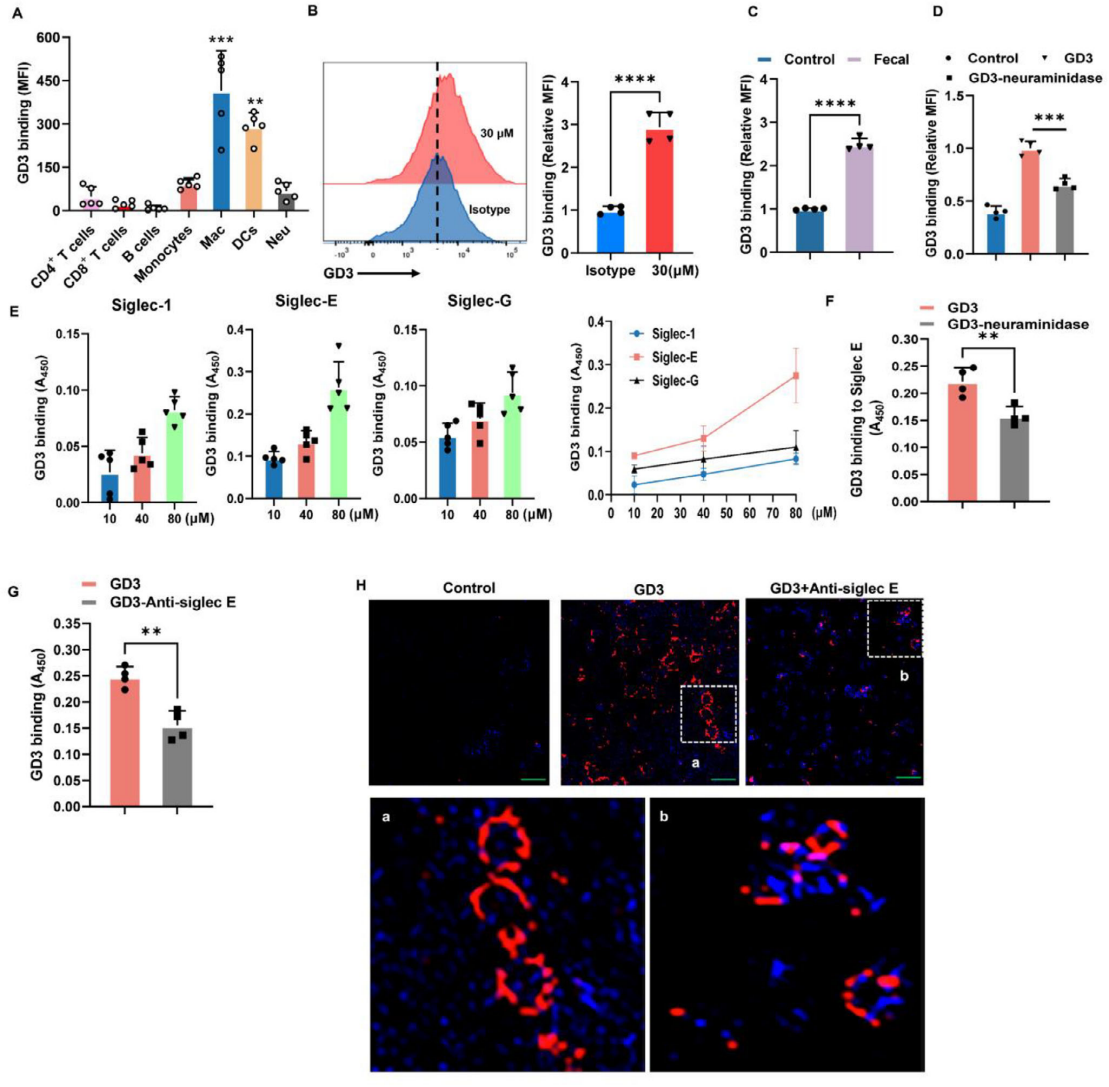
GD3 binds to Siglec‐E expressed by macrophages through sialic acid. A,B) Binding of GD3 to T cells, B cells, monocytes, macrophages, DCs, neutrophils from LI‐LPL (A) and macrophages derived from bone marrow (BMDMs) (B). LPL or BMDMs were incubated with biotinylated GD3 (30 µm), then analyzed by flow cytometry; results are presented as the fold changes of mean fluorescence intensity. C,D) Flow cytometry analysis of the binding of the DMSO control, GD3 (C), or neuraminidase‐treated GD3 (D) to BMDMs treated with fecal contents isolated from mice with colitis. E) Binding of different concentrations of GD3 to recombinant proteins of Siglecs. F) Binding of GD3 or neuraminidase‐treated GD3 to recombinant Siglec‐E protein. G) Binding of GD3 to recombinant Siglec‐E protein in the absence of or presence of anti‐Siglec‐E mAb. H) Fluorescence microscopy of Raw264.7 cells incubated with biotinylated GD3 and streptavidin‐phycoerythrin (Red) with/without anti‐Siglec‐E mAb. Original magnification, ×200. Scale bar represents 100 µm. Statistical comparisons were performed using a two‐tailed unpaired *t*‐test (B,F); one‐way ANOVA with Tukey's multiple comparisons test (A,D,E). Error bars indicate mean ± SD. *p < 0.05, **p < 0.01, ***p < 0.001, ****p < 0.0001. *n* = 5 (A,E) or *n* = 4 (B–D,F,G) independent biological samples.

We questioned whether the higher levels of St8sia1 and its derived ganglioside GD3 are linked to the production of colonic T_reg_ cells. To investigate if GD3 interacts with immune cells, we isolated LPLs from the colon and incubated the cells with biotinylated GD3. GD3 highly bound to primary macrophages compared to other immune cells (**Figure** **4**A). This binding was also found in BMDMs (Figure 4B) and the Raw 264.7 macrophage cell line (Figure S4A, Supporting Information). The binding increased over time in Raw 264.7 cells, with maximum binding occurring at 4 h, and with increasing concentrations of GD3 at 60 µm (Figure S4B, Supporting Information). Interestingly, this binding was greatly enhanced in BMDMs or Raw 264.7 cells that were pretreated with fecal content from mice with colitis (Figure 4C; Figure S4C, Supporting Information). However, these interactions were significantly reduced after GD3 was treated with neuraminidase to remove sialic acid in BMDMs (Figure 4D) and Raw264.7 cells (Figure S4D,E, Supporting Information). These data demonstrated that the binding was dependent upon sialic acid. Siglecs predominantly bind to sialic acids of cell surface proteins and lipids. We first tested a panel of Siglec recombinant proteins for their interaction with GD3. Indeed, GD3 bound recombinant Siglec‐1, Siglec‐E, and Siglec‐G, with the greatest binding seen for Siglec‐E at 80 µm (Figure 4E). However, neuraminidase treatment partially decreased GD3 binding to recombinant Siglec‐E, but not Sigle‐G and Siglec‐1 (Figure 4F; Figure S4F, Supporting Information). Moreover, a blocking antibody to Siglec‐E (anti‐Siglec‐E) inhibited the binding of GD3 to recombinant Siglec‐E (Figure 4G) and macrophages (Figure 4H). Siglec‐E is considered a murine functional homolog of human Siglec‐9. We found GD3 and GM3 can bind to human Siglec‐9‐Fc. However, they have less ability to be binding to Siglec‐9‐Fc compared to Siglec‐E‐Fc (Figure S4G, Supporting Information). Together, these data indicate that GD3 is a sialic acid‐containing glycolipid ligand to Siglec‐E on macrophages.

### 2.4 GD3/Siglec‐E Binding Induces Metabolic Reprogramming and Controls an Anti‐Inflammatory Phenotype in Macrophages that Facilitates the Generation of T_reg_ Cells

**Figure 5:**
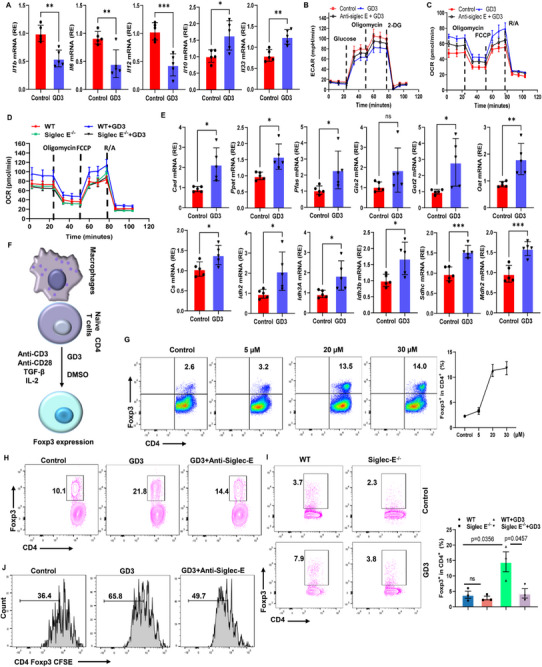
GD3‐Siglec E binding induces metabolic reprogramming in macrophages that promotes the generation of T_reg_ cells. A) Real‐time PCR analysis of the indicated genes in BMDM treated with fecal contents for 17 h in the absence or presence of 20 µm GD3. B) ECAR in BMDMs treated with fecal contents for 17 h in the presence of DMSO (Control), 20 µm GD3 or GD3+ anti‐Siglec‐E mAb. C) OCR in BMDMs treated with fecal contents for 17 h in the presence of DMSO (Control) or 20 µm GD3 or GD3+ anti‐Siglec‐E mAb. D) OCR in WT and Siglec‐E^−/−^ BMDMs treated with fecal contents for 17 h in the presence of DMSO (Control) or 20 µm GD3. E) Real‐time PCR analysis of the genes related to glutaminolysis and TCA cycle in BMDM treated with fecal contents for 17 h in the absence or presence of 20 µm GD3. F) Coculture: naive CD4^+^ T cells (1 × 10^5^) were cocultured with macrophages (1 × 10^5^) in suboptimal T_reg_ cell‐inducing conditions (1 ng mL^−1^ transforming growth factor (TGF)‐β, 1 µg mL^−1^ CD3 antibody, 100 U mL^−1^ IL‐2) and analyzed on day 6 by flow cytometry. G) Frequencies of Foxp3^+^ CD4^+^ T cells after naive CD4^+^ T cells were cocultured with splenic macrophages exposure to various concentrations of GD3 as described in (F). H,I) The Siglec E on macrophages is required for GD3 T_reg_ cell‐inducing effects. Naive CD4^+^ T cells were cocultured with WT BMDMs (H) or Siglec‐E^−/−^ BMDMs (I) in the presence (H) or absence (I) of anti‐Siglec E mAb. J) Assessment of cell proliferation. Naive CD4^+^ T cells were labeled with CFSE and cultured with BMDMs as in (F), in the presence of GD3 (20 µm). CFSE dilution in CD4^+^Foxp3^+^ cells was assessed on day 4 by FACS. The percentage shows the cells that underwent the indicated number of cell divisions. Statistical comparisons were performed using a two‐tailed unpaired *t*‐test (A,E); one‐way ANOVA with Tukey's multiple comparisons test (G,I). Error bars indicate mean ± SD. *p < 0.05, **p < 0.01, ***p < 0.001, ****p < 0.0001. *n *= 3 independent biological samples.

To better explore the functional changes in macrophages after GD3/Siglec‐E ligation, bone marrow cells were differentiated into macrophages for 7 days with M‐CSF in the presence or absence of GD3, followed by treatment with lipopolysaccharide (LPS) and IFN‐γ or by treatment with fecal contents. The differentiated macrophages showed lower expression of *Il1b*, *Il6*, and *Il12*, and higher expression of *Il10* and *Il33* mRNA relative to differentiated BMDMs in the absence of GD3, indicating GD3 treatment induces M2‐like macrophages (**Figure** **5**A). To investigate whether GD3‐treated human macrophages adopt an anti‐inflammatory phenotype via Siglec‐9, we differentiated the human THP‐1 monocytic leukemia immortalized cell line to macrophage using phorbol 12‐myristate 14‐acetate (PMA). Differentiated cells were then treated for 2–3 days with a combination of cytokines including IL‐4 and transforming growth factor β (TGF‐β) in the presence of GD3 with or without anti‐Siglec‐9 antibody. GD3 treatment greatly enhances the IL‐4/TGF‐β‐mediated polarization of macrophages toward an anti‐inflammatory state, as evidenced by the lower levels of *Il1b*, *Il6*, and *Il12* mRNA. However, these effects were reversed by an anti‐Siglec‐9 antibody (Figure S5A, Supporting Information). As cellular metabolism and polarization of macrophages are mutually linked,^[^ ^22^ ^]^ we then investigated these GD3‐induced metabolic changes in macrophages. Extracellular flux analysis showed that GD3 directly suppressed the fecal contents‐induced extracellular acidification rate, the glycolytic capacity and glycolytic reserve capacity in macrophages after a stimulation of 17 h (Figure 5B; Figure S5B, Supporting Information). However, GD3‐treated macrophages showed an enhanced mitochondrial OCRs, ATP production, and an increased spare respiratory capacity (Figure 5C; Figure S5C, Supporting Information), indicating GD3 induced the higher level of fatty acid oxidation contributed to polarization of macrophages. Importantly, these significant changes in phenotype were rescued in part by blocking antibody to Siglec‐E (Figure 5B,C; Figure S5B,C, Supporting Information) or deletion of Siglec‐E in macrophages (Figure 5D), indicating the GD3/Siglec‐E signal regulates macrophage metabolism. We next carried out real‐time PCR analysis to assess the effects of GD3 treatment on BMDMs. BMDMs exposed to GD3 showed increased expression of several genes related to glutaminolysis genes including *Cad, Ppat, Pfas, Gls2, Got2*, and *Oat*. Furthermore, metabolic enzymes in the TCA cycle including citrate synthase (Cs), isocitrate dehydrogenase (*Idh2, Idh3*), succinate dehydrogenase complex subunits (*Sdhc*), malate dehydrogenase (*Mdh2*) were also increased after GD3 treatment (Figure 5E), suggesting that GD3/Siglec‐E may support oxidative phosphorylation (OXPHOS) for the generation of M2 type macrophages.

To assess whether macrophages were required for GD3‐mediated potentiation of T_reg_ cell induction, we next co‐cultured CD11b^+^ macrophages from the lamina propria (LP), splenic macrophages, or BMDMs with naive CD4^+^ T cells in the presence or absence of GD3 under T_reg_ differentiation conditions (Figure 5F). GD3‐treated splenic macrophages (Figure 5G), BMDMs (Figure S5D, Supporting Information), or LP macrophages (Figure S5E, Supporting Information) significantly enhanced T_reg_ cell frequencies and increased the expression of IL‐10 in T_reg_ cells (Figure S5F, Supporting Information). However, when naive CD4^+^ T cells were activated by beads coated anti‐CD3/CD28 antibodies, the addition of GD3 failed to increase T_reg_ cell frequencies and proliferation (Figure S5G, Supporting Information), indicating GD3 cannot induce the generation of T_reg_ cells directly. Since the interaction of Siglecs with their ligands is dependent on sialic acid and can be disrupted by sialidase,^[^ ^18^ ^]^ blocking of Siglec‐E in the coculture of macrophages with CD4^+^ T cells reduced Foxp3 induction in response to GD3 (Figure 5H). In addition, macrophages lacking Siglec‐E (Siglec‐E KO) fail to generate a higher frequency of Foxp3^+^ cells by the addition of GD3 (Figure 5I). To follow T‐cell proliferation in coculture, naïve CD4^+^ T cells were stained with carboxyfluorescein succinimidyl ester (CFSE) before incubation with BMDMs. Four days later, T cells were stained for Foxp3 expression and analyzed for CFSE dilution. GD3‐treated macrophages induced the development of a higher number of proliferating Foxp3^+^ CD4^+^ cells (Figure 5J) or CD4^+^ T cells (Figure S5H, Supporting Information); however, these effects were reduced by the addition of anti‐Siglec‐E antibody (Figure 5J; Figure S5H, Supporting Information). These results support our finding that GD3 acts upon macrophages to potentiate the induction of T_reg_ cells and suggest that Siglec‐E is involved in this process.

### 2.5 GD3 Binding‐Induced IL‐33 Production in Macrophages Contributes to the Proliferation of T_reg_ Cells

**Figure 6:**
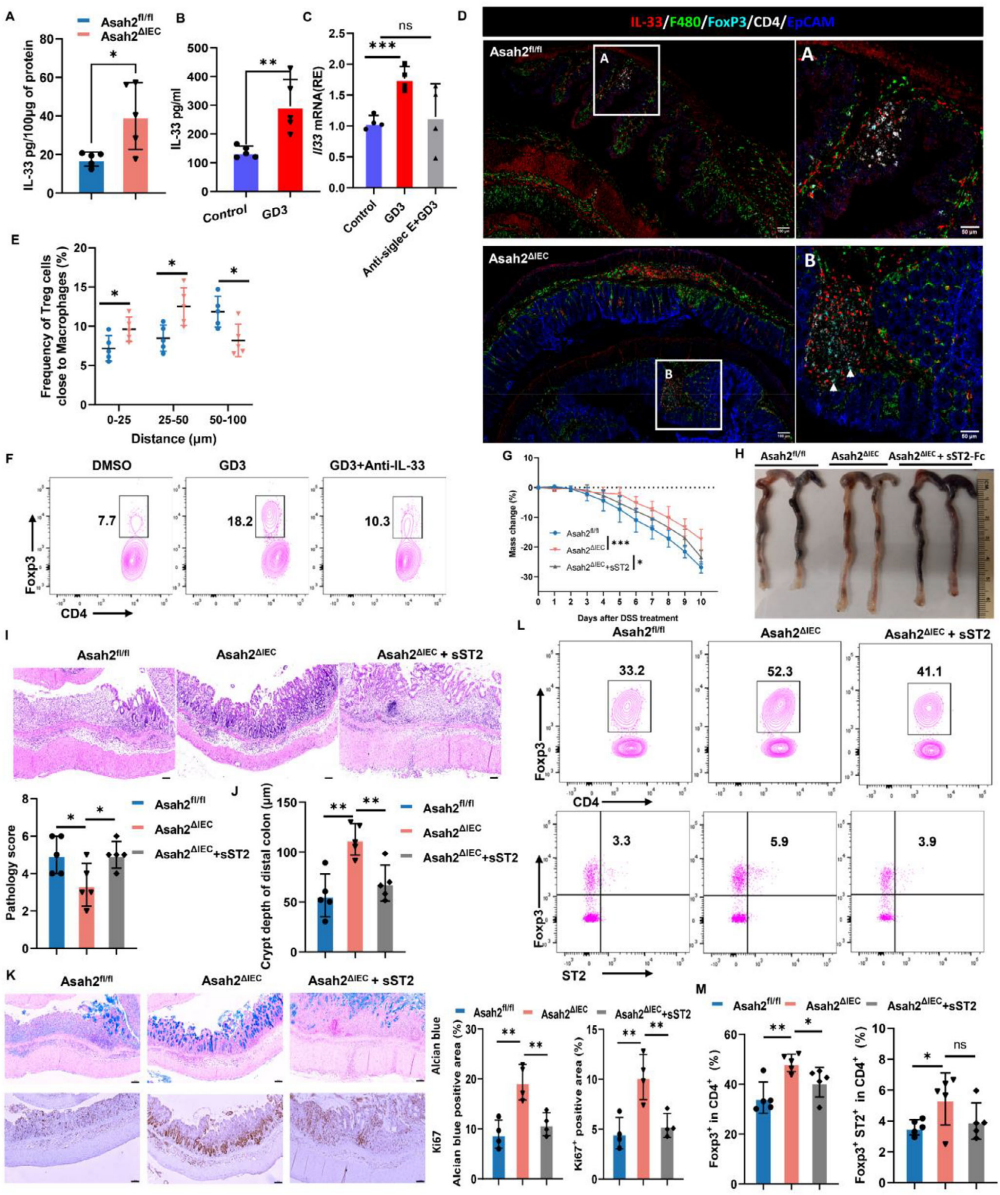
GD3‐Siglec‐E ligation induces macrophages to secrete IL‐33 associated with colitis prevention via the induction of ST2^+^ T_reg_ cells. A) ELISA analysis of IL‐33 in colon tissues from DSS‐treated *Asah2^ΔIEC^* and *Asah2^fl/fl^* mice (6 days post‐DSS treatment). B) Quantification of IL‐33 in the supernatant from naive CD4^+^ T cells cocultured with macrophages in suboptimal T_reg_ cell‐inducing conditions as experiment in Figure 5F. *n* = 5 independent biological experiments. C) Real‐time PCR analysis of Il33 mRNA expression in Raw 264.7 cells treated with control vehicle, GD3 (20 µm), or GD3+ anti‐Siglec E mAb. D) Representative IMC images of three independently stained colon samples of colon tissues from DSS‐treated of *Asah2^ΔIEC^* and *Asah2^fl/fl^* mice with the protein panel colored by different markers. A magnification of the indicated region (white box) from the images is shown on the bottom. Marker expression was false colored, and markers are indicated above each plot. A Gaussian blur (sigma = 0.65) was applied. Scale bars, 50 µm. The arrow indicates the IL‐33^+^ macrophage‐T_reg_ cell interaction. E) Quantification of the distance between IL‐33^+^ macrophage‐T_reg_ cell interaction in colon sections analyzed from DSS‐treated mice from each strain. An unpaired two‐tailed *t*‐test was used for statistical analysis. F) Frequencies of Foxp3^+^ CD4^+^ T cells after naive CD4^+^ T cells were cocultured with BMDMs exposure to 20 µm GD3 in the presence or absence of anti‐IL‐33 mAb as described in Figure 5F. G–M) IL‐33 was blocked during the treatment of DSS via sSt2. PBS or 100 µg sST2 was injected i.p. into *Asah2^ΔIEC^* on days 4, 5, 6, 7, and 8 of the DSS challenge phase. G) Weight loss in animals following the induction of colitis, measured as a reduction from initial weight until the day of sacrifice. Data are from five independent experiments; two‐way repeated‐measures ANOVA followed by Tukey's posttest. H) Colon length images. I) Representative histology staining of the colon and histological score. J) Crypt depth of the distal colon. K) Alcian blue staining and immunohistochemistry staining of Ki67 in the colon. L,M) Flow cytometry analysis of Foxp3^+^ T_reg_ and ST2^+^Foxp3^+^ T_reg_ cells in the colon. Statistical comparisons were performed using two‐tailed unpaired *t*‐test (A,B); one‐way ANOVA with Tukey's multiple comparisons test (C,I–K,M). Error bars indicate mean ± SD. *p < 0.05, **p < 0.01, ***p < 0.001, ****p < 0.0001. *n *= 5 (A,B) or *n* = 4 (K,M) independent biological samples. Scale bar represents 100 µm.

Our CyToF data showed that DSS‐treated *Asah2^ΔIEC^* mice have higher numbers of ST2^+^ T_reg_ cells in the colon. The IL‐33/ST2 pathway plays a pivotal role on the proliferation and accumulation of Foxp3^+^ T_reg_ cells.^[^ ^23^ ^]^ To further analyze how GD3 enhances TGFβ‐mediated differentiation of T_reg_ cells, we assayed for cytokines released from colon tissues and cocultures of macrophage‐CD4 cells. Despite similar induction of IL‐17A and IFN‐γ in colon tissues isolated from DSS‐treated *Asah2^ΔIEC^* and *Asah2^fl/fl^* mice (Figure S6A, Supporting Information), IL‐33 release was increased in colon tissues isolated from DSS‐treated *Asah2^ΔIEC^* mice compared with *Asah2^fl/fl^* mice (**Figure** **6**A). In addition, we used an ELISA to confirm that GD3 induced higher secretion of IL‐33 in the cocultures of macrophage‐CD4 cells (Figure 6B). We also treated BMDMs with fecal contents obtained from wild‐type mice with DSS‐induced colitis in the presence or absence of GD3. Of note, the addition of GD3 induced the highest level of *Il33* mRNA expression in BMDMs (Figure S6B, Supporting Information) or Raw264.7 cells (Figure 6C) in the presence of fecal contents. These significant changes in Il33 expression were inhibited in part by blocking antibody to Siglec‐E (Figure 6C; Figure S6B, Supporting Information). It is known that IECs‐derived factors, for example, TGF‐β and retinoic acid (RA),^[^ ^24^, ^25^, ^26^ ^]^ could participate in conferring the tolerogenic phenotype upon macrophage/dendritic cells, which were able to promote T_reg_‐cell differentiation. To evaluate whether IECs‐derived GD3 can directly “educate” macrophages, we isolated the colonic IECs from WT (WT‐IEC) and Asah2^ΔIEC^ (KO‐IEC) mice with DSS‐induced colitis. BMDMs were cocultured with IECs and treated by the lysate of fecal contents in the presence or absence of anti‐GD3 antibody for 24 h. The BMDMs cocultured with KO‐IEC showed higher expression of *Il33* relative to BMDMs cocultured with WT‐IEC. However, this effect was inhibited by the presence of anti‐GD3 antibody in the coculture (Figure S6C, Supporting Information). Together, these data indicate GD3/Siglec‐E signal contributes to IL‐33 secretion in macrophages.

We then explored the colonic lymphocyte compartment of DSS‐treated *Asah2^ΔIEC^* and *Asah2^fl/fl^* mice to look at the interactions between macrophages and T_reg_ cells by using imaging mass cytometry (IMC). T_reg_ and macrophages were localized in the inflammatory region of the colon in both *Asah2^ΔIEC^* and *Asah2^fl/fl^* littermate control mice after DSS exposure (Figure 6D). However, more macrophages in the colon of *Asah2^ΔIEC^* mice were seen in close proximity to T_reg_ cells compared to *Asah2^fl/fl^* mice (Figure 6D,E). In particular, we found a higher proportion of IL‐33^+^ macrophages within 50 µm of T_reg_ cells in the colons of *Asah2^ΔIEC^* mice, while IL‐33^+^ macrophages were found at a distance greater than 50 µm from T_reg_ cells in the colons of *Asah2^fl/fl^* mice (Figure 6E). To confirm IL‐33 is responsible for the proliferation of T_reg_ cells, we next cocultured BMDMs with naive CD4^+^ T cells in the presence of GD3 with/without anti‐IL‐33 mAb under T_reg_ differentiation conditions. GD3 treatment induced Foxp3 expression (Figure 6F) and promoted the proliferation of CD4 T cells or Foxp3^+^ cells (Figure S6D, Supporting Information), however, these effects were reduced by the addition of anti‐IL‐33 antibody (Figure 6F; Figure S6D, Supporting Information).

Having observed that IL‐33^+^ macrophages play a prominent role in colonic T_reg_ induction in colitis and that soluble ST2 (sST2) can act as a decoy receptor that can block the binding of IL‐33 to ST2, we next questioned whether IL‐33 blockade through sST2 treatment would reverse the phenotypes in DSS‐induced *Asah2^ΔIEC^* mice. We treated *Asah2^ΔIEC^* with sST2 injected intraperitoneally to block IL‐33 signaling on days 4, 5, 6, 7, and 8 following DSS challenge and assessed whether sST2 reduced the generation of T_reg_ cells and reversed the protection of *Asah2^ΔIEC^* mice from colitis. *Asah2* ^ΔIEC^ mice treated with sST2 displayed significantly increased signs of disease which included weight loss (Figure 6G), colon shortening (Figure 6H; Figure S6E, Supporting Information), rectal bleeding, and blood in stools (Figure S6F,G, Supporting Information) compared with DSS‐treated *Asah2* ^ΔIEC^ mice that received an isotype control. Consistently the histological score (Figure 6I), crypt depth (Figure 6J), goblet cell area, and Ki67^+^ cells (Figure 6K) were reduced upon treatment with sST2 when compared with PBS‐treated *Asah2* ^ΔIEC^ mice. In addition, sST2‐treated mice showed increased *Il1b* and *Il6* and reduced *Muc2* and *ZO1* mRNA expressions upon DSS challenge when compared with PBS‐treated *Asah2* ^ΔIEC^ animals (Figure S6H, Supporting Information). Blockade of IL‐33 signaling also led to the decreased percentage of T_reg_ cells in the colon of *Asah2* ^ΔIEC^ mice on day 10 following DSS exposure, which is like that seen in wild‐type mice (Figure 6L,M). Thus, these data reveal an essential role of GD3‐IL‐33 signaling in macrophages in promoting T_reg_ cell proliferation in the colon.

### 2.6 St8sia1 Deficiency Promotes DSS‐Induced Colitis in Mice

**Figure 7:**
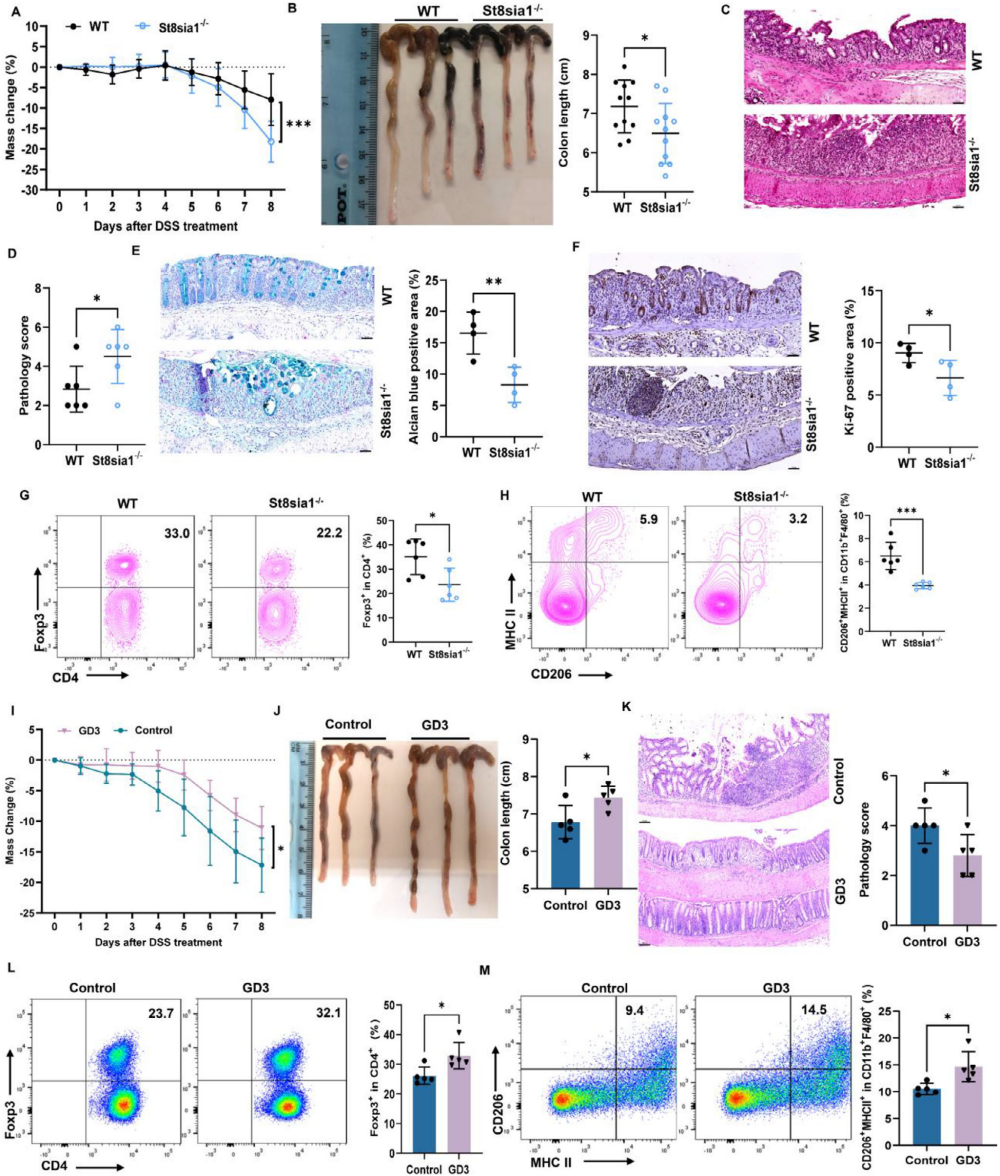
St8sia1 and its metabolite GD3 control the development of colitis. A–H) WT and *St8sia1* ^−/−^ mice were treated with 2.5% DSS in drinking water for 8 days. A) Weight loss in animals following the induction of colitis, measured as a reduction from initial weight until the day of sacrifice. Data are from two independent experiments: two‐way repeated‐measures ANOVA followed by Tukey's posttest. B) Colon images and length. C,D) Representative histology staining of colon and histological score. Scale bar represents 50 µm. E) Alcian blue staining of colon. Scale bar represents 50 µm. F) Immunohistochemistry staining of Ki67 in the colon. Scale bar represents 50 µm. G,H) Flow cytometry analysis of Foxp3^+^ T_reg_ (G) and CD11b^+^F4/80^+^MHCII^+^CD206^+^ (H) macrophages in the colon. I–M) C57BL/6J mice were fed 10 mg of extracted GD3 per day at 1 week before and after DSS administration. I) Weight loss in animals following the induction of colitis, measured as a reduction from initial weight until the day of sacrifice. Data are from two independent experiments: two‐way repeated‐measures ANOVA followed by Tukey's posttest. J) Colon images and length. K) Representative histology staining of colon and histological score. Scale bar represents 100 µm. L,M) Flow cytometry analysis of the percentage Foxp3^+^ T_reg_ (L) and CD11b^+^F4/80^+^MHCII^+^CD206^+^ (M) in the colon. Statistical comparisons were performed using a two‐tailed unpaired *t*‐test; Error bars indicate mean ± SD. *p < 0.05, **p < 0.01, ***p < 0.001. *n* = 5 (G,H,L,M) independent biological samples. Scale bar represents 100 µm.

We next investigated the influence of St8sia1 and its product GD3 on the induction of mucosal inflammation after DSS exposure. Notably, St8sia1^−/−^ mice featured enhanced susceptibility to DSS‐induced colitis compared to WT littermates, manifesting as an exacerbated weight loss (**Figure** **7**A), colon shortening (Figure 7B), rectal bleeding, and blood in stools (Figure S7A,B, Supporting Information), on day 8 after the start of DSS supplementation. The epithelial damage, including mucosal erosion, lymphocyte infiltration, and crypt loss, was also increased in the colons of St8sia1^−/−^ mice (Figure 7C,D; Figure S7C, Supporting Information). The goblet cells, Ki67^+^ cells, and occludin^+^ cells were reduced in the colons of St8sia1^−/−^ mice when compared with these in WT mice (Figure 7E,F; Figure S7D, Supporting Information). Importantly, the T_reg_ cell population and CD206^+^ macrophages in the lamina propria were significantly less in St8sia1^−/−^ mice than in control mice (Figure 7G,H; Figure S7E, Supporting Information).

We thus hypothesized that increased amounts of dietary GD3 may influence T_reg_ proliferation in the gut and prevent colitis. To test this, C57BL/6J mice were orally given with 0.25% of a GD3 diet calculated by weight (10 mg mouse^−1^ day^−1^) every day starting 1 week prior to DSS exposure. After treated with 10 mg of the GD3 diet per day, mice had the reduction in weight loss (Figure 7I), colon shortening (Figure 7J), rectal bleeding and blood in stools (Figure S7F,G, Supporting Information) and less colonic pathology and crypt loss (Figure 7K; Figure S7H, Supporting Information), and increased goblet cells (Figure S7I, Supporting Information) and IL‐33 secretion in the colon tissue (Figure S7J, Supporting Information) on day 12 post DSS treatment compared with control mice. Consistently, there was a higher percentage of T_reg_ cells and ST2^+^Foxp3^+^ cells (Figure 7L; Figure S7K, Supporting Information) and CD11b^+^F4/80^+^CD206^+^ macrophages (Figure 7M) in the LPLs of mice treated with GD3. Real‐time PCR analysis and immunofluorescent staining found that GD3 treatment reduced the expression of several genes related to pro‐inflammatory cytokines (*Il6*, *Il1b*, and *Tnfa*) and increased the expression of genes and proteins related to mucins and tight junction, including *Muc3*, *ZO1*, and *Claudin‐1* (Figure S7L,M, Supporting Information). Together, these data indicated that the St8sia1 and metabolite GD3 might regulate colitis and modulate the T_reg_ cell population in the lamina propria.

## 3 Discussion

Intestinal epithelial glycosylation is altered during colitis.^[^ ^4^ ^]^ Abnormal mucus sialylation disrupts host‐microbial interactions, mucin barrier function, and mucosal immunity.^[^ ^10^, ^27^ ^]^ It is not clear how the sialic acid‐containing lipids regulate gut homeostasis. In this study, we showed that IEC‐specific deletion of NcDase decreased susceptibility to DSS‐induced colitis in mice and affected the ganglioside profiles in the intestine. An increase in the number of T_reg_ cells was induced by epithelial GD3‐treated macrophages in the colonic lamina propria. GD3‐Siglec‐E signaling on macrophages promoted the IL‐33‐dependent proliferation of T_reg_ cells. St8sia1^−/−^ mice showed a significantly severe phenotype of DSS‐induced colitis compared to that of wild‐type mice. Thus, the orchestration of ceramidases and sialyltransferases controls mucosal immunity which influences IBD.

NcDase is highly expressed in the intestinal epithelium and catalyzes the hydrolysis of ceramide^[^ ^28^ ^]^ which can be used for the synthesis of glucosylceramide. Our findings suggest that epithelial NcDase exerts critical effects on maintaining the intestinal ganglioside GD3 by the regulation of St8sia1. Recently, several studies have shown an association between glucosylceramide, gangliosides, and IBD.^[^ ^12^, ^29^, ^30^ ^]^ Reduction of GD3 species is associated with the pathogenesis of colitis. The content of ganglioside catabolic enzymes beta‐hexosaminidase and sialidase is elevated in patients with UC and CD.^[^ ^12^ ^]^ Sialyltransferases transfer sialic acid to glycans on proteins and lipids.^[^ ^5^ ^]^ Mice carrying a St6galnac1 mutation, the dominant sialyltransferase specifically expressed in goblet cells, have compromised mucus barriers, dysbiosis, and susceptibility to intestinal inflammation.^[^ ^10^, ^31^ ^]^ Our findings demonstrate that the NcDase‐St8sia1/GD3 axis is critical for dampening pro‐inflammatory signals leading to IBD. It should notice that both GD3 and its precursor GM3 were upregulated in Asah2^ΔIEC^ mice, raising the possibility that GM3 may also contribute to the phenotype. GM3 was reported to act as pro‐ and anti‐inflammatory, which are dependent on the species of ceramide including acyl‐chain length (16‐24) and modifications (α‐hydroxylation, ω‐9 desaturation).^[^ ^32^ ^]^ TLR4 activation is selectively enhanced by very‐long‐chain (VLCFA, 22:0; 24:0)‐GM3 species, but suppressed by long‐chain fatty acids (LCFA, 16:0;18:0, 20:0)‐ and unsaturated VLCFA (ω‐9)‐GM3 species. However, the specific logical functions of these species GM3 36:1, GM3 42:2, GD3 42:0, and GD3 42:2 in our study are unclear. Future studies should investigate whether these individual GM3 species interact with Siglecs and have pro‐ or anti‐inflammatory activities in vivo.

IECs play a critical role in controlling the activity of immune cells in the gut. Our results emphasize that IEC‐derived GD3 can drive the development of “tolerogenic mucosal” macrophages, as demonstrated by the higher expression of CD206 and IL‐33 in cells treated with GD3 and in LP‐macrophages from *Asah2* ^ΔIEC^ mice. Aberrant glycosylation is a hallmark of colitis that often results in decreased sialylation,^[^ ^4^ ^]^ which has been shown to result in the engagement of Siglecs. We have identified Siglec‐E as a myeloid‐cell‐modulating receptor of GD3. T_reg_ cells are critical for continued immune tolerance in the intestine, and their disruption is a primary cause of IBD.^[^ ^33^ ^]^ Published reports have demonstrated that CX3CR1^−^CD103^+^CD11b^+^ DCs induce T_reg_ cells and that this function is dependent on TGF‐β and retinoic acid.^[^ ^34^ ^]^ We found that more CD206^+^ CD11b^+^ macrophages reside adjacent to the colonic T_reg_ cells in *Asah2^ΔIEC^* mice. It has been reported that T‐cell differentiation requires distinct GSL types for activation, which implies that control of GSL expression would offer a strategy targeting specific T‐cell populations to treat immune diseases.^[^ ^35^ ^]^ Previous studies have shown that GSLs increase the proportion of T_reg_ cells in experimental autoimmune encephalomyelitis.^[^ ^36^ ^]^ However, in our study, we observed that GD3 failed to increase T_reg_ cell frequencies when naive T cells were activated by beads coated with CD3/CD28 antibodies, suggesting a T cell‐extrinsic immunoregulatory mechanism that is distinct from those reported for other GSLs. Our data showed that the binding of sialylated lipid by macrophage was shown to enhance the induction of T_reg_ through engagement of Siglec‐E, which can be reversed by blockade of GD3‐Siglec‐E binding in the presence of anti‐Siglec‐E mAb or deletion of Siglec‐E in macrophage. These macrophages with an M2‐like phenotype were able to promote the proliferation and activation of T_reg_ cells. The increase in the proliferation of T cells seen in our system did appear to be due to the upregulation of IL‐33 expression on ‘GD3‐treated’ macrophages, as this was inhibited by the IL‐33 blocking antibody. The finding that the binding of GD3 to Siglec‐E increased expression of IL‐33 by macrophages was an important observation, as IL‐33 is an important link between inflammation‐driven tissue damage and the local intestinal T_reg_ cell response.^[^ ^23^ ^]^ We demonstrated here that the GD3/IL‐33 axis induced T_reg_ cell proliferation which led to the amelioration of DSS‐induced colitis. Published studies have demonstrated that IL‐33 can be derived from myeloid cells^[^ ^37^ ^]^ and is involved in the maintenance and accumulation of T_reg_ cell populations in the colonic lamina propria,^[^ ^23^ ^]^ consistent with our results. Our results showed that the pathogenesis of colitis induced by DSS was exacerbated in St8sia1^−/−^ mice, which was associated with decreased T_reg_ accumulation. These results suggested that the St8sia1/GD3‐Siglec‐E axis decreased inflammatory responses to DSS‐induced epithelial cell damage as a result of the proliferation of T_reg_ cells in the colon. These findings might reflect the interactions between macrophages and naïve CD4^+^ T cells in the hyposialylated gut microenvironment in IBD. Thus, the Siglec‐sialoglycan axis of immune modulation might be an important mediator of sialic acid‐induced immune suppression in the context of colitis.

In conclusion, our study showed that epithelial NcDase‐related metabolite GD3 plays a major role in controlling macrophage function by conferring upon them a tolerogenic “mucosal” phenotype. IEC‐macrophages can induce the development of colonic T_reg_ cells with potent suppressor activity that protects against experimental colitis. This could open new avenues for possible therapeutic intervention with GD3 treatment.

## 4 Experimental Section

### Mice

C57BL/6 mice, B6(Cg)‐Siglece^tm1.2Avrk^/J mice (Stock No: 032008), and B6.Cg‐Tg(Vil1‐cre)1000Gum/J mice (Vil‐Cre 1000, Stock No: 021504) were obtained from Jackson Laboratory. B6;129S‐St8sia1^tm1Rlp^/Mmmh mice (MMRRC_000037_MU) (St8sia1^−/−^) were obtained from NIH MMRRC and were backcrossed at least 4 generations to C57BL/6. Genotyping of St8sia1^−/−^ mice was screened with primers: GD3S‐9692: 5’‐CACAGTTACATCTACATGCCT‐3’; GD3S‐9694: 5’‐GCAAGACGTTGTCATAGTAGT‐3’; RLP290‐Neo: 5’‐ TCGCCTTCTTGACGAGTTCTTCTGAG‐3’. Products from PCR screening were: Wild type: ≈320 bp; Heterozygous: ≈320 bp, ≈220 bp; Homozygous knockout: ≈220 bp. Asah2^fl/fl^ mice were generated by CRISPR‐Cas9‐targeted insertion of LoxP recombination sites into intronic regions that surrounded exons 4–5 (Biocytogen). The single‐guide RNA (sgRNA) sequences were: sgRNA‐2: 5′‐ACAGTCCAGTGCATAACACTGGG‐3’; sgRNA‐14: 5′‐GCCTGCAGTGATGATATAGCTGG‐3’. Genotyping of Asah2^fl/fl^ mice was screened with primers ASAH2‐F2: 5′‐ AATGCCTGTAAGGAACCAATTGAGG‐3′; ASAH2‐R2: 5′‐ CTGCCTTAATTTTGGAAGGTGACCC‐3′. Products from PCR screening were: Wild type: ≈353 bp; Mutant: ≈438 bp. Asah2^fl/fl^ mice were crossed with Villin‐Cre mice (B6.Cg‐Tg(Vil1‐cre)997Gum/J mice, RRID: IMSR_JAX:004586, The Jackson Laboratory) for generating mice with IEC‐specific deletion of Asah2 (designated as Asah2^ΔIEC^). Asah2^fl/fl^ mice were crossed with Asah2^ΔIEC^ to generate control Asah2^fl/fl^ mice and Asah2^ΔIEC^ littermates. St8sia1^−/−^ mice, Asah2^ΔIEC^ mice, and their littermates were used for experiments. All animal studies were approved by the University of Louisville Institutional Animal Care and Use Committee (23256).

### DSS‐Induced Colitis Models

Experimental colitis was induced in 8‐ to 12‐week‐old male and female WT mice and littermate controls by the addition of 2.5% (wt/vol) dextran sulfate sodium (DSS) (36–50 KD molecular weight, MP Biomedicals, OH) in their drinking water for 8–12 days. Body weight was monitored daily. For GD3 treatment (Avanti Polar Lipids), mice were orally given 10 mg kg^−1^ body weight per day for one week before colitis induction and maintained every day after DSS treatment. The amount (10 mg kg^−1^ body weight per day) of dietary milk GD3 was calculated based on previous reports,^[^ ^14^ ^]^ which showed that in individuals who consumed 1 g of milk fat fraction containing ≈43 mg day^−1^ ganglioside (80% GD3; 20% GM3 [w/w]) for 8 weeks showed an improved intestinal integrity. For recombinant mouse soluble ST2 Fc chimera protein treatment, mice were treated *i.v*. with 10 µg sST2‐Fc protein (1004‐MR‐050, R&D Systems) on days 4–8 after DSS treatment. For tissue macrophage depletion, mice were injected *i.v*. with 200 µL of clodronate liposomes (Encapsula Nanosciences) on day 0, 2, and 4 after DSS treatment.

### Scoring of the DSS‐Induced Colitis Models

The pathological severity of induced colitis was assessed blindly by an experienced pathologist using a scoring system for DSS colitis, based on tissue damage and inflammatory cell infiltration. Briefly, each sample was graded semi‐quantitatively from 0 to 3 for tissue damage, the scoring was defined as follows: 0, no mucosal damage; 1, isolated focal epithelial damage; 2, extended epithelial damage associated with mucosal erosions, crypt loss, and ulcerations; 3, extensive damage deep into the bowel wall. For infiltration of inflammatory cells, the scoring was defined as follows: 0, no signs of inflammation; 1, increased presence of inflammatory infiltrate in the mucosa; 2, submucosal presence of inflammatory cell clusters; 3, transmural extension of inflammatory infiltrations. Pathology scores included scores from each criterion in tissue damage and infiltration of inflammatory cells. Total scores were added to give a maximum score of 6.^[^ ^38^ ^]^

For bleeding scores, the scoring was determined by fecal Hemoccult single slides (Beckman Coulter) as follows: 0, hemoccult negative; 1, hemoccult positive; 2, visible traces of stool blood; 3, gross rectal bleeding.

For stool scores, the scoring was determined as follows: 0, normal stools; 1, semi‐formed stools without adhering to the anus; 2, semi‐formed stools adhering to the anus; 3, liquid stools adhering to the anus.^[^ ^39^ ^]^

### Histology and Immunohistochemistry

Intestinal tissues were flushed with PBS, coiled into “Swiss rolls”, fixed in 10% formalin, dehydrated, and then embedded in paraffin. Tissue samples were cut at 5 µm thicknesses and stained with hematoxylin and eosin or alcian blue. For immunohistochemistry (IHC) studies, tissue sections were stained with the following antibodies: anti‐Occludin (BS‐1495R, Bioss), anti‐ haptoglobin (HP) (BS‐1808R, Bioss), and anti‐Ki‐67 (1:200, clone SolA15, Thermo Fisher Scientific). Antigen retrieval was performed in citrate buffer (pH 6.0) for 30 min at 95 °C. After antigen retrieval, sections were cooled for 30 min followed by blocking solution for 1 h at room temperature. Tissue sections were stained with primary antibodies overnight at 4 °C. After washing, HRP‐conjugated secondary antibodies were incubated for 1 h at room temperature, followed by DAB (Vector Laboratories) staining for 30 min. Tissue sections were counterstained with hematoxylin for 3 min. Sections were scanned using a PANNORAMIC DESK II DW scanner (3D Histech) and the area with immunohistochemically positive staining within the field of interest was calculated by the Image J software after setting the thresholds.

### Preparation of Intestinal Epithelial Cells (IECs) and Lamina Propria Lymphocytes (LPLs)

The method used for epithelial cell preparations from the intestine was reported previously.^[^ ^3^, ^40^ ^]^ In brief, fat tissues, feces, and Peyer's patches (PPs) were removed from the intestine, and the intestine was opened longitudinally and rinsed with PBS. The intestine was cut into 0.5‐ to 1‐cm‐long small pieces and incubated in 10 mL HBSS solution supplemented with 5 mm EDTA and 10 mm HEPES for 10 min at 37 °C with shaking at 180 rpm. This process was repeated for a total of 2 rounds. Supernatants were pooled and washed in HBSS with 10 mm HEPES at 300 g for 6 min at 4 °C. Cell pellets constituted isolated intestinal epithelial cell fractions.

For LPLs preparations from mice, the intestine was processed as above to remove the IECs. Tissues were then incubated at 37 °C rocking at 180 rpm for 25 min in 5 mL RPMI supplemented with 10% FBS, 10 mm HEPES, 0.08 mg mL^−1^ DNase I (Roche), and 1 mg mL^−1^ collagenase type IV (Worthington). This process was repeated for a total of 2 rounds. Finally, the solution containing digested tissue was passed through a 70 µm cell strainer, and the cells were collected. After centrifugation, the cells were further isolated on a Percoll gradient (40%/72%; GE Healthcare) at 2300 rpm for 20 min; the LPLs were recovered at the interface of the 40% and 72% Percoll solutions. For flow cytometry analysis, the cells were labeled using standard procedures described below.

### Reagents, Antibodies, and Flow Cytometry

Splenocytes and LPLs were incubated with anti‐CD16/32 (Catalog# 14‐0161‐85, Thermo Fisher Scientific) to prevent nonspecific antibody binding before staining with appropriate surface antibodies for 30 min at 4 °C, washed with PBS+2% FCS, and used for FACS analysis. Intracellular staining of Foxp3 was performed using the Foxp3 Fix/Perm Buffer Set (eBioscience, Thermo Fisher). For detection of intracellular cytokines, cells were first stimulated for 5 h with 50 ng mL^−1^ PMA and 1 µg mL^−1^ ionomycin in the presence of brefeldin A (5 µg mL^−1^; all obtained from Sigma), followed by staining for surface markers. Cells were then fixed and permeabilized using the Foxp3 Fix/Perm Buffer Set and stained for intracellular cytokines. The following antibodies were used at a dilution of 1/100–1/1000: FITC or APC‐labeled anti‐Foxp3 (FJK‐16s, eBioscience, Thermo fisher), PerCP‐Cy5.5, PE‐, FITC‐ or APC‐labeled anti‐IL‐17A (TC11‐18H10.1, 1:100), PE‐ or APC‐labeled anti‐F4/80 (BM8, Biolegend, 1:200), PE‐ or APC‐labelled anti‐CD206 (MR6F3, 1:200), PE‐, FITC‐ or APC‐labeled anti‐CD4 (RM4‐5, 1:300), PE‐Cy7‐labeled anti‐CD3 (145‐2C11, 1:100), APC‐ or PE‐Cy7‐labeled anti‐IFN‐γ (XMG1.2, 1:200), PE‐ or FITC‐labeled anti‐mouse Ly6G (1A8, 1:200), FITC‐, PerCP‐Cy5.5 or Pacific Blue‐labelled anti‐CD45 (30‐F11, 1:400), and PE, PerCP‐Cy5.5, FITC‐ or APC‐CD11b (M1/70, Biolegend, 1:500); PerCP‐Cy5.5‐labeled anti‐MHCII (M5/114.15.2, 1:1000); PE‐ or APC‐labelled anti‐CD103 (2E7, 1:200); PerCP‐Cy5.5‐labeled anti‐ST2 (RMST2‐2, 1:200): PE‐labelled anti‐GATA3 (TWAJ, 1:200); FITC‐labelled anti‐Helios (22F6, 1:300); PerCP‐Cy5.5‐labeled anti‐CD8 (53‐6.7, 1:300), APC‐labeled anti‐NK1.1 (PK136, 1:200), APC‐labeled anti‐B220 (RA3‐682, 1:300), PE‐labeled anti‐LY6C (HK1.4, 1:300), APC‐labeled anti‐CD11c (N418, 1:400), PerCP‐Cy5.5‐labeled anti‐CD44 (IM7, 1:200), and PE‐labeled anti‐CD62L (MEL‐14, 1:300). All antibodies were obtained from ThermoFisher unless otherwise noted. Flow cytometry data were acquired on a 5‐color FACScan (Becton Dickinson) and analyzed using FlowJo v10.10.0. Cell sorting was performed using a FACSAria II.

### Time‐Of‐Flight Mass Cytometry (CyTOF) Experiments

Single cell suspensions of LPLs were centrifuged at 350 × g at 4 °C for 5 min and then resuspended in 1 mL PBS containing 0.1% BSA, 2 mm EDTA , and 0.05% sodium azide. Primary metal‐labeled antibody staining was performed on ice for 40 min at a dilution of 1:100, unless otherwise noted. The metal‐labeled antibodies included: IFNγ (165Ho), IL‐5 (143Nd), IL‐33R (156Gd), IL‐17A (165Yb), Foxp3 (158Gd), TCRgd (154Sm), Ly6G (141 Pr), CTLA (154Sm), CD11c (142 Nd), CD8 (168Er), CD11b (172Yb), CD19 (149 Sm), F4/80 (146 Nd), CD206 (169Tm),), PD‐1 (159Tb), CD3e (152 Sm), CD274 (153Eu), CD62L (160 Gd), CD25 (151 Eu), Ly6c (150Nd), NK1.1 (170 Er), Cx3cr1 (164 Dy), CD103 (163 Dy), CD44 (162Dy), CD68 (147Sm), CD4 (145Nd), MHCII (209Bi), and B220 (176 Yb). Following primary antibody staining, cells were washed, blocked with a commercial Fc‐blocking reagent, and resuspended in 0.5 mL PBS with a 1:4000 dilution of cisplatin viability reagent (Cell‐ID Cisplatin‐ Fluidigm, Ca) for 5 min at room temperature. Cells were washed twice for terminating cisplatin staining using the staining buffer. The samples were analyzed on a Helios Mass Cytometer (Fluidigm, South San Francisco, CA). The resulting FCS files were normalized using a bead‐based normalization algorithm in the CyTOF acquisition software. FCS files were manually pre‐gated on 193Ir DNA^+^CD45^+^ events to exclude cisplatin‐positive dead cells, doublets, and DNA‐negative debris.

### Isolation of Antigens from Fecal Content for In Vitro Cell Culture

Fresh fecal content was collected and carefully resuspended in PBS. The obtained suspension was centrifuged at 400 g for 5 min to remove larger particles. Suspensions were then lysed by physical disruption through sonication. The protein concentration of the lysate was quantified using a Bradford protein assay. Total fecal content (20—50 µg mL^−1^) was used for in vitro stimulation.

### In Vitro Bone Marrow‐Derived Macrophages (BMDMs) Differentiation

Bone marrow cells were flushed from the femur and tibia bones of female 8‐week‐old mice and were differentiated for 7 days in the presence of recombinant mouse macrophage colony‐stimulating factor (M‐CSF; 20 ng mL^−1^; PeproTech) in complete medium (RPMI‐1640 medium containing 10 mm glucose, 2 mm L‐glutamine, 100 U mL^−1^ of penicillin‐streptomycin, and 10% FBS). BMDMs on day 7 were washed and then stimulated for 24 h with lipopolysaccharide (LPS, 10 ng mL^−1^; Sigma), IFN‐g (100 ng mL^−1^; R&D Systems), or fecal supernatant and GD3 (20 µm) in the presence or absence of anti‐Siglec E (10 µg mL^−1^). BMDMs were then harvested and performed by XF‐96 Extracellular Flux Analyzer for Seahorse real‐time cell metabolic analysis or were cocultured with Naive (CD44^−^ CD62L^+^) CD4^+^ T cells.

### Cell Culture Experiments

Naive (CD44^−^ CD62L^+^) CD4^+^ T cells, splenic macrophages, and LPL macrophages (CD11b^+^ F4/80^+^) were sorted by FACS from C57BL/6 mice. Macrophages (1 × 10^5^) plus naive CD4^+^ T cells (1 × 10^5^) cocultures were set up in the presence of 5 µg mL^−1^ of monoclonal CD3ε antibody and 2 µg mL^−1^ of monoclonal CD28 antibody (InvivoMAb, BioXcel). GD3 (Cat#860060P) ganglioside was purchased from Avanti Polar Lipids (Alabaster, AL) and used at 5–30 µm. For T‐cell activation in the absence of macrophages, naive CD4^+^ T cells (1 × 10^5^) were incubated with mouse T‐activator CD3/CD28 Dynabeads (11456D, Thermofisher) at a 1‐to‐1 bead‐to‐cell ratio. Suboptimal T_reg_‐induction conditions consisted of 1 ng mL^−1^ recombinant mouse TGF‐β1 (PeproTech) and 100 U mL^−1^ recombinant IL‐2 (Cat# 212‐12, PeproTech). All in vitro polarization assays were carried in complete RPMI with 10% FBS (final volume of 200 µL) in round‐bottom 96‐well plates (USA Scientific). For assessment of cell proliferation, BMDMs (2 × 10^5^ or 5 × 10^5^) were cocultured with 5 µm Celltrace CFSE‐labeled naïve CD4^+^ T (1 × 10^5^) in the presence of anti‐CD3 antibody, anti‐CD28 antibody, and IL‐2 (100 U mL^−1^) in 200 µL RPMI complete medium in 96‐well round‐bottom plates. Ninety‐six hours after plating the cells were incubated with PMA and ionomycin for 1 hour, followed by adding the protein transport inhibitor brefeldin (Cat# 00‐ 4506–51, Thermo Fisher Scientific) for 5 h after which they were collected and were processed for staining for flow cytometry.

To investigate the role of GD3 on the differentiation and polarization of macrophages, bone marrow cells were incubated with GD3 and differentiated in the presence of M‐CSF. For blocking binding with anti‐Siglec E, BMDMs were treated with 10 µg mL^−1^ anti‐Siglec E or isotype control before being incubated with GD3 for 4 h at 4 °C. To inhibit the action of IL‐33, 200 ng mL^−1^ antibody to IL‐33 was added.

To investigate whether GD3‐treated human macrophages adopt an anti‐inflammatory phenotype via Siglec‐9, the human THP‐1 monocytic leukemia immortalized cell line was differentiated to macrophage using phorbol 12‐myristate 14‐acetate (PMA, 25 ng mL^−1^). Differentiated cells were then treated for 2–3 days with a combination of cytokines including IL‐4 (20 ng mL^−1^ and (TGF‐β) (50 ng mL^−1^) in the presence or absence of GD3 with or without human anti‐Siglec‐9 antibody (MAB1139, Novus Biologicals).

### Oxygen‐Consumption Rate (OCR) and Extracellular Acidification Rate (ECAR)

BMDMs were plated in seahorse XF‐96 well cell culture microplates at a density of 1 × 10^5^ cells per well and left untreated or treated with GD3 (20 µm) for 24 h. BMDMs were rested for 45–60 min in XF assay medium at 37 °C in a non‐CO_2_ incubator before running the Seahorse XFe96. XF‐96 Extracellular Flux Analyzer (Seahorse Bioscience) was used for real‐time measurements of BMDMs ECAR and OCR. Three or more consecutive ECAR measurements were obtained under basal conditions and after the sequential addition of 10 mm glucose, 1.5 µm oligomycin, and 50 mm 2‐DG (2‐deoxyglucose). In the OCR assay, three or more consecutive measurements were obtained under basal conditions and after the sequential addition of 1.5 µm oligomycin, 1.5 µm FCCP (fluoro‐carbonyl cyanide phenylhydrazone), and 0.5 µm rotenone plus 0.5 µm antimycin A.

### Immunofluorescence and Image Mass Cytometry (IMC)

For immunofluorescence analysis, OCT (Sakura Finetek)‐embedded tissue cryosections (5 µm‐thick) were blocked for 1 hour at 22 °C with 5% BSA in DPBS and incubated overnight at 4 °C with the primary antibodies, that is, St8sia1 (1:200, 24918‐1‐AP, Thermo Fisher Scientific), Ki‐67 (1:200, clone SolA15, Thermo Fisher Scientific), Occludin (1:100, BS‐1495R, Bioss) and EpCAM(1:200, clone 1B7, Thermo Fisher Scientific). Primary antibodies were detected by Alexa Fluor 488, 594, or 647 conjugated goat anti‐mouse, anti‐rabbit IgG, and anti‐rat IgG (1:600, Invitrogen). The TUNEL assays were performed using an in situ Apoptosis Detection Kit (Invitrogen) according to the manufacturer's instructions. Tissues were counterstained with 4’,6‐diamino‐2‐phenylindole (DAPI) staining and images were captured on a Zeiss LSM 510 confocal microscope equipped with Zen blue imaging software (Zeiss). For IMC, slides were washed in the DPBS and incubated with 1:500 Cell‐ID Intercalator‐Ir (Fluidigm) in DPBS for 10 min and rinsed with MilliQ water before air‐drying overnight. Some antibody clones were obtained in purified format and conjugated in house using MaxPar conjugation kits (Fluidigm) according to the manufacturer's protocol. Some antibody clones conjugated with metal isotopes were purchased from Fluidigm where available (mouse CyTOF catalog). IMC images were acquired using a Hyperion Imaging Mass Cytometer. To enhance visualization of the small lymphocytes, the channels for IL‐33, Foxp3, CD206, EpCAM, CD4, and F4/80 were filtered using a bandpass Fast Fourier Transformation. The relative distribution of cell types within the tissues compared between the two genotypes was estimated using marginal means calculation. Each region of interest was selected such that it would contain an area with intestinal damage including adjacent normal tissue where possible. For IMC images, Fiji ImageJ v2.0.0 was used to make composite images of selected channels. Six images obtained from three mice were selected for this study, ranging from 1–9 mm.^[^ ^2^ ^]^

### Binding of GD3 to Cells

1 × 10^6^ isolated LPLs, BMDMs and RAW264.7 cells were incubated for the indicated times on ice with 30 µm of the appropriate biotinylated GD3 ganglioside (#27202, Cayman) in DMSO including 0.5% BSA in the presence or absence of anti‐Siglec E (10 µg mL^−1^) or GD3 without sialylation (that was treated with 0.15 U mg^−1^ neuraminidase (NA) for 1 h at 37 °C). After incubation with fluorescent conjugates of streptavidin, cells were washed and analyzed by flow cytometry or fluorescent microscopy.

### Binding of GD3 to Recombinant Siglecs

96‐well plates were coated with recombinant mouse Siglec (1, E and G) fusion proteins (1 µg mL^−1^) in 50 mm carbonate/bicarbonate buffer, pH 9.5, overnight at 4 °C. Wells were blocked with ELISA buffer at room temperature for 2 h. After incubation with the indicated concentrations of biotinylated GD3 in the presence or absence of anti‐Siglec E (10 µg mL^−1^) for 2 h at 37 °C, the plates were washed 4 times with ELISA buffer. The biotinylated GD3 was detected by horseradish peroxidase (HRP)‐conjugated streptavidin and substrate, then the absorbance at 450 nm was recorded. GD3 without sialylation was treated with 0.15 U mg^−1^ neuraminidase (NA) for 1 h at 37 °C.

### Cytokine Analysis

The quantity of IL‐33, IL‐17A, and IFN‐γ (Thermo Fisher, eBioscience) was determined in culture supernatants and tissue using an enzyme‐linked immunosorbent assay (ELISA) kit according to the manufacturer's instructions. The sensitivity of the assays was <20 pg mL^−1^. Mouse colon supernatant samples were also evaluated for cytokine levels using the U‐PLEX mouse cytokine 19‐plex kit from Meso Scale Discovery (MSD, Cat. No. K15069M‐1). The MSD multiplex assay plates were precoated with capture antibodies. Samples for analysis or kit standards were added at a volume of 50 µL per well after pre‐diluting the original sample with assay diluent. The plates were washed after a two‐hour incubation at room temperature with agitation. Sulfo‐tagged detection antibodies were added and incubated for another two hours at room temperature with agitation. Following the incubation, the plates were washed once again. 2× Read Substrate was added and plates were read on the MSD reader. All data were analyzed by MSD Discovery Workbench Software 4.0.

### Gangliosides Analysis

Intestine samples were weighed to ≈50 mg, then 1 mL of ultrapure water was added, and samples were spiked with 20 microliters of GM1 (36:1)‐d3 (10 ng µL^−1^). Samples were homogenized in a Fisher bead mill tissue homogenizer using ceramic beads. Homogenates were transferred to 13 mL glass centrifuge tubes and subjected to biphasic (Svennherholm) extraction using chloroform, methanol, and water. Each sample was extracted twice, and the upper aqueous phases from each extraction were pooled. Samples were dried in a Speedvac concentrator and resuspended in 200 microliters of methanol, then transferred to LC‐MS vials containing small‐volume glass inserts. The LC‐MS platform consisted of a Dionex Ultimate 3000 HPLC coupled to a Thermo LTQ Orbitrap XL mass spectrometer. The LC system included two pumps, a vacuum degassing system, an autosampler, and a column oven. The HPLC column was a Phenomenex 2 mm × 100 mm HILIC column (3 micron,100 Angstrom pore size) equipped with a guard cartridge of the same column chemistry. LC solvents were Solvent A: ultrapure water containing 50 mm ammonium formate, and Solvent B: 95:5 acetonitrile:50 mm ammonium formate. The flow rate was 200 microliters per minute, and the column oven was held at 25 °C. The autosampler was held at 4 °C. Twenty microliters of each sample was injected. The gradient conditions used were: Time 0–3 min, 100% solvent B. From time 3 to 15 min, Solvent B was linearly ramped to 50%, then held at 50% B for 5 min. The HILIC column was then re‐equilibrated at the starting conditions for 10 min. The column eluent was diverted to waste for the first 5 min of the LC run. Column eluent was introduced to a Thermo LTQ‐Orbitrap XL mass spectrometer via a heated electrospray ionization source. The mass spectrometer was operated in negative ion mode at 60 000 resolution with full scan MS data collected from 600–2000 m/z. Study samples were analyzed using full MS mode only. Data‐dependant product ion spectra were collected on pooled samples to fragment the 4 most abundant ions at 7500 resolutions using the FT analyzer. The electrospray ionization source was maintained at a spray voltage of 4.5 kV with sheath gas at 30 (arbitrary units) and auxiliary gas at 10 (arbitrary units). The inlet of the mass spectrometer and the heated ESI source were maintained at 350 °C.

### Data Analysis

Chromatographic alignment, isotope correction, peak identification, and peak area calculations were performed using El‐MAVEN software (Elucidata). Concentrations of each analyte were determined against the peak area of the deuterated internal standard. The total amount of each analyte in each sample was normalized to the number of cells in each sample. Confirmed analytes were identified by comparison of aligned LC‐MS peaks to the median m/z values, retention times (RT), and MS/MS fragmentation spectra of authentic ganglioside reference standards.

### RNA Extraction and PCR

Total RNA extractions from tissues or cells were performed using the RNeasy Mini kit (Qiagen) and converted into cDNAs with the Superscript IV reverse transcriptase (Thermo Fisher Scientific) according to the manufacturer's instructions (Thermo Fisher Scientific). Real‐time (RT) PCR was carried out in the ABI Quant Studio Real‐Time (RT) PCR system using cDNA as template with SYBR Green Master Mix (Invitrogen) and the primers listed in Table S1 (Supporting Information). The fold changes in mRNA expression were determined by the 2^−(ΔΔCt)^ method after their normalization to the housekeeping gene *GAPDH* or *β‐actin* as an internal control, which was set at a value of 1. Differences between groups were determined using a two‐sided Student's *t*‐test or ANOVA. Error bars on plots represent ± SD. All primers were purchased from Sigma.

### Western blot Analysis

IECs were disrupted in RIPA lysis buffer with protease and phosphatase inhibitors (Roche) for 30 min on ice. Protein lysates were quantitated using a Bio‐Rad protein kit (Bio‐Rad), 100 µg of lysates were separated on 10% SDS polyacrylamide gels and transferred to polyvinylidene difluoride (PVDF) membranes (Bio‐Rad). The membranes were blocked with 5% BSA in Tris‐buffered saline (20 mm Tris‐HCl, pH 7.4; 150 mm NaCl) with 0.05% Tween 20 (TBST) for 1 h at room temperature (RT). Primary antibodies for St8sia1 (1:1000, ThermoFisher Scientific) and GAPDH (1:4000, Sigma) were incubated at 4 °C overnight. Following four washes with TBST for 5 min each, HRP‐conjugated secondary antibodies were incubated for 1 h at RT. After four washes with TBST for 5 min each, the membranes were treated using the Enhanced Chemiluminescent (ECL) reagent (ThermoFisher Scientific). The images were acquired with the ChemiDoc MP System (Bio‐Rad).

### Gene Expression Profiling by RNA‐Seq

Colonic IECs were isolated from DSS‐treated mice as described above. Total RNA was extracted using Trizol reagent (Thermo Fisher, 15596018) and purified using Dynabeads Oligo (dT) (Thermo Fisher) with two rounds of purification and checked with Agilent Technologies 2100 Bioanalyzer for RNA integrity. A Poly(A) RNA sequencing library was prepared following Illumina's TruSeq‐stranded‐mRNA sample preparation protocol. The mRNA was fragmented into short fragments using divalent cations under elevated temperature (94 °C) (NEB, cat. e6150) and the cleaved RNA fragments were reverse transcribed to cDNA by SuperScript II Reverse Transcriptase (Invitrogen, cat. 1896649). Paired‐ended sequencing was performed on Illumina's NovaSeq 6000 sequencing system. The reads containing sequencing adaptors, sequencing primers, and sequences with a Q quality score lower than 20 were removed prior to assembly. The cleaned sequencing reads were aligned to the reference genome using the HISAT2 package, which built a database of potential splice junctions. Multiple alignments with a maximum of two mismatches were allowed for each read sequence. StringTie was used for assembling the aligned reads of individual samples. Transcriptomes from all samples were then merged to reconstruct a comprehensive transcriptome using a proprietary Perl script of LC Sciences (Houston, Texas, U.S.A.). FPKM reads and differential expressed genes were evaluated by StringTie and edgeR, respectively. The differentially expressed mRNAs and genes were selected with log2 (fold change) ≥ 1 or log2 (fold change) ≤ −1, and with *p‐*values < 0.05.

### Quantification and Statistical Analysis

Values were shown as Mean ± SD except where otherwise indicated. Prism (GraphPad Software, version 9.2.0) was used to determine statistical significance. One‐way ANOVA with Tukey's test, two‐way ANOVA with Sidak's test, two‐tailed unpaired *t‐*test, and two‐tailed Pearson's *r* correlation were used for analysis. The asterisks indicated significant differences. *p < 0.05, **p < 0.01, *** *p* < 0.001, **** *p* < 0.0001. n represents the number of independent samples.

## Conflict of Interest

The authors declare no conflict of interest.

## Author Contributions

Z.D. and Z.X. designed the study, analyzed and interpreted the data, and prepared the manuscript. Z.X., C.L., M.K.S., T.W., A.T., and X.S. performed the experiments and interpret the data. G.D., Y.T., and C.J.M. interpreted the findings.

## Supporting information



Supporting Information

## Data Availability

The data that support the findings of this study are available from the corresponding author upon reasonable request.
