# Supplementary material for: Intestinal Neutral Ceramidase Deficiency Triggers Regulatory T Cell Response via Gd3 to Protect the Host from Intestinal Inflammation
Source: Adv Sci (Weinh). 2025 Nov 7;13(5):e12681. doi: 10.1002/advs.202512681 (PMC12849859; doi:10.1002/advs.202512681)
Supplement: Supplementary file 1 — Supporting Information [file ADVS-13-e12681-s001.docx]

**Supplementary Table S1. Primers used for Real-time PCR**

| Gene name | Forward primer | Reverse primer |
| --- | --- | --- |
| *Gapdh* | AGGTCATCCCAGAGCTGAACG | ACCCTGTTGCTGTAGCCGTAT |
| *β-actin* | ACGGCCAGGTCATCACTATTC | AGGAAGGCTGGAAAAGAGCC |
| *IL-1β* | GCAACTGTTCCTGAACTCAACT | ATCTTTTGGGGTCCGTCAACT |
| *IL-6* | TAGTCCTTCCTACCCCAATTTCC | TTGGTCCTTAGCCACTCCTTC |
| *IL-10* | GCTCTTACTGACTGGCATGAG | CGCAGCTCTAGGAGCATGTG |
| *IL-12a* | TACTAGAGAGACTTCTTCCACAACAAGAG | TCTGGTACATCTTCAAGTCCTCATAGA |
| *IL-33* | TCCAACTCCAAGATTTCCCCG | CATGCAGTAGACATGGCAGAA |
| *Tnf-α* | TCTATGGCCCAGACCCTCAC | GACGGCAGAGAGGAGGTTGA |
| *Muc2* | ATGCCCACCTCCTCAAAGAC | GTAGTTTCCGTTGGAACAGTGAA |
| *Muc3* | GCCGTGAATTGTATGAACGGA | CGCAGTTGACCACGTTGACTA |
| *Cxcl9* | TCCTTTTGGGCATCATCTTCC | TTTGTAGTGGATCGTGCCTCG |
| *Ccl4* | TTCCTGCTGTTTCTCTTACACCT | CTGTCTGCCTCTTTTGGTCAG |
| *Cldn1* | GACTGTTGATGATGGTTATCGG | AGATGGTAAGGTACAGCCAAGG |
| *Ocln* | TTGAAAGTCCACCTCCTTACAGA | CCGGATAAAAAGAGTACGCTGG |
| *Zo-1* | TGGGCAAGGGATAGGAGTG | ATATGGCTGGCCAATCGA |
| *St8sia1* | GCTACCCGTAGGAGCCAGT | CAGCACCCCTTGCACAATCT |
| *St8sia5* | AGATTTGTTGGGGAATCGAACTT | GCTGTCATTAAAGAGCCCAGTC |
| *Ugcg* | GGAATGGCCTTGTTCGGCT | CGGCTGTTTGTCTGTTGCC |
| *Gba* | GCCAGGCTCATCGGATTCTTC | CACGGGGTCAAGAGAGTCAC |
| *Ugt8a* | ACTCCATATTTCATGCTCCTGT | AGGCCGATGCTAGTGTCTTGA |
| *B3galt4* | TCTGGACCCTGTTTGGACCTT | GGCCGTACACACCAGGATG |
| *B4galt6* | GGGTCTCCAATCGCTCTCTG | ATAAAGAGGTACGTGTTGGCG |
| *B4galnt1* | GCCCTCTATGCGCTAGTCTTG | GTGCTGGAGTACAGGAGACC |
| *St3gal5* | ATGCCAAGTGAGTTCACCTCT | ACTCCAAATGCAACCAACGTG |
| *Cad* | CTGCCCGGATTGATTGATGTC | GGTATTAGGCATAGCACAAACCA |
| *Ppat* | GCGAGGAATGTGGTGTGTTTG | TTTAGGCACTGCACTCCCATC |
| *Pfas* | GACTCCAGCATCGACCAACAT | GAAAGTCGGTAACGTCGGGT |
| *Gls2* | CAGAGGGACAGGAGCGTATC | TTCTTTCGGAATGCCTGAGTC |
| *Got2* | GGACCTCCAGATCCCATCCT | GGTTTTCCGTTATCATCCCGGTA |
| *Oat* | GGAGTCCACACCTCAGTCG | CCACATCCCACATATAAATGCCT |
| *Cs* | GGACAATTTTCCAACCAATCTGC | AGTCAATGGCTCCGATACTGC |
| *Idh2* | GGAGAAGCCGGTAGTGGAGAT | GGTCTGGTCACGGTTTGGAA |
| *Idh3a* | TGGGTGTCCAAGGTCTCTC | CTCCCACTGAATAGGTGCTTTG |
| *Idh3b* | TGGAGAGGTCTCGGAACATCT | AGCCTTGAACACTTCCTTGAC |
| *Mdh2* | TTGGGCAACCCCTTTCACTC | GCCTTTCACATTTGCTCTGGTC |
| *Sdhc* | GCTGCGTTCTTGCTGAGACA | ATCTCCTCCTTAGCTGTGGTT |

**
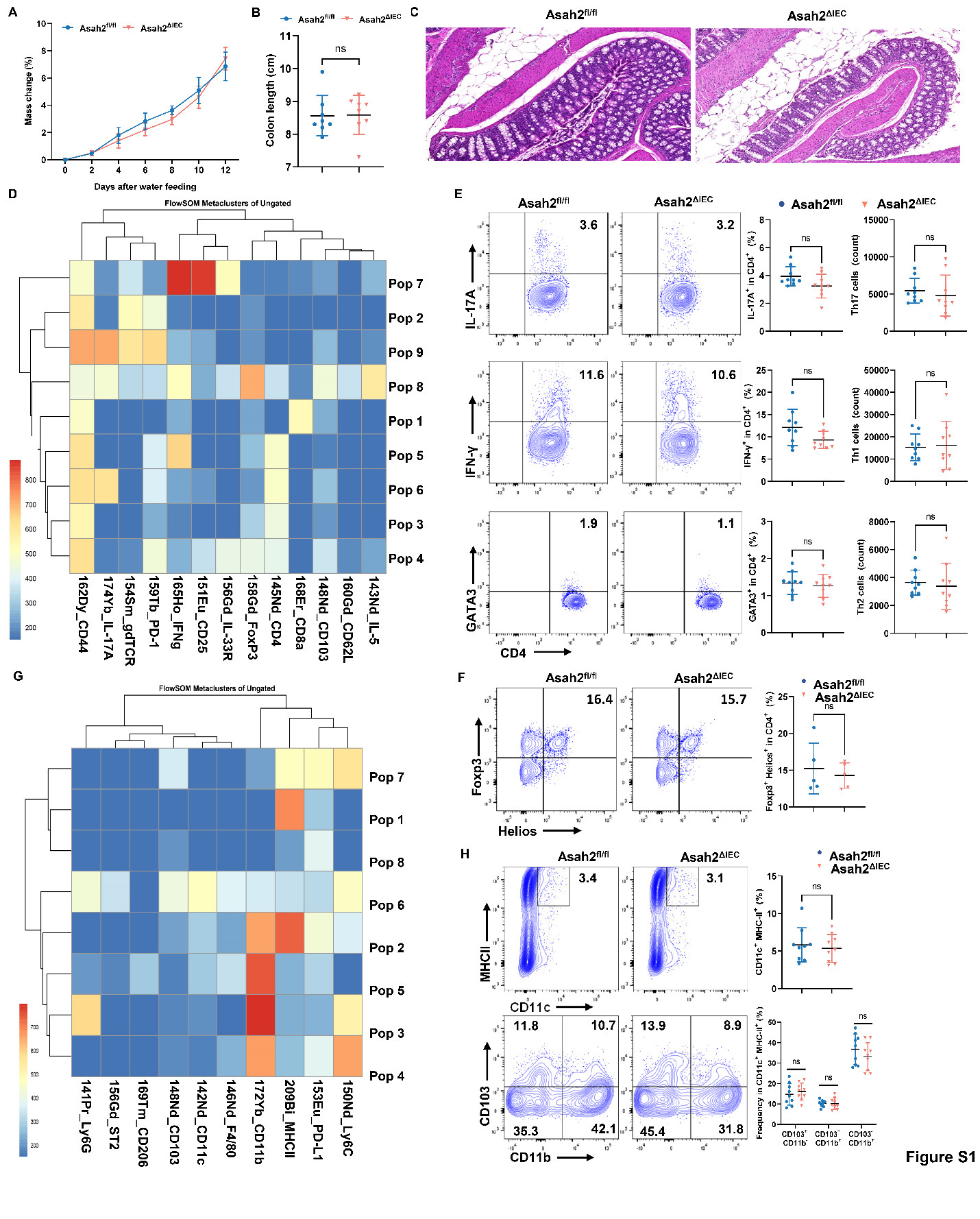
Figure S1. Analysis of immune profiles within colon based on CyTOF analysis.**

*Asah2^ΔIEC^* and *Asah2^fl/fl^* mice were treated with water (**A-C**) or 2.5% DSS in drinking water (D-H) for 12 days.

(**A**) Changes in percent of body weight at the indicated days post-water treatment.

(**B**) Colon length.

(**C**) Representative histology staining of colon.

(**D**) An in-depth T cell population analysis using FlowSOM clustering annotated into T cell subtypes is shown as a normalized expression heatmap. Data are representative of five independent experiments.

(**E**) Flow cytometry analysis of the percentage and number of Th17, Th1 and Th2 cells in the colon.

(**F**) Flow cytometry analysis of the percentage and number of Helios^+^Foxp3^+^ T_reg_ cells in the colon.

(**G**) An in-depth myeloid cells population analysis using FlowSOM clustering annotated into CD11b cell subtypes is shown as a normalized expression heatmap. Data are representative of five independent experiments.

(**H**) Flow cytometry analysis of the percentage of CD11c^+^MHCII^+^ DCs and CD11c^+^MHCII^+^CD11b^+^CD103^+^ DCs in the colon.

Statistical comparisons were performed using two-tailed unpaired t-test. Error bars indicate mean ± SD. Each dot represents one mouse (E, F, H).


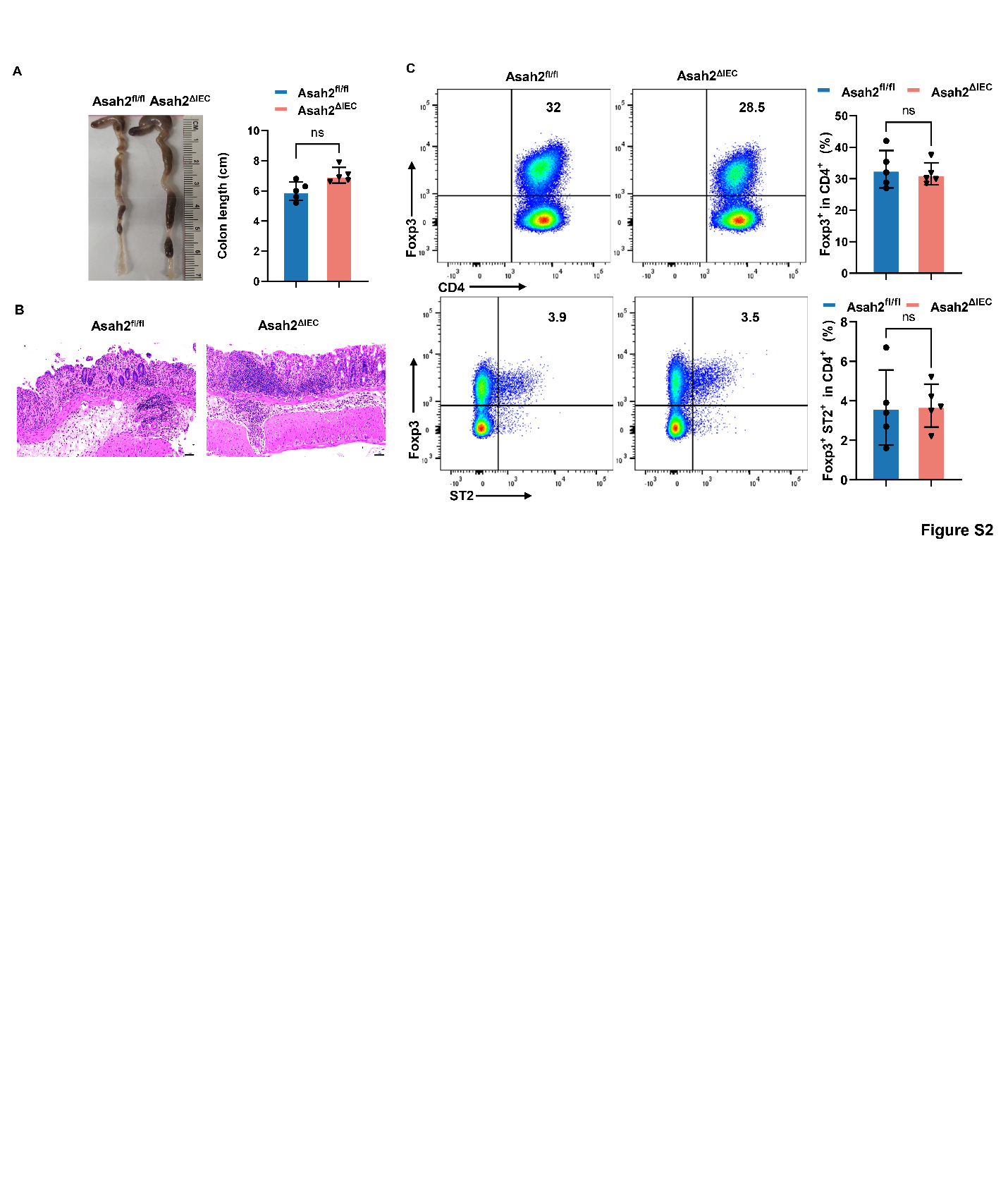


**Figure S2** **Macrophages are crucial for protecting against intestinal inflammation and inducing T_reg_ cells in DSS-treated *Asah2^ΔIEC^* and *Asah2^fl/fl^* mice.**

*Asah2^ΔIEC^* and *Asah2^fl/fl^* mice were treated with 2.5% DSS in drinking water for 12 days and treated with clodronate liposomes (Clodrosome).

(**A**) Colon images and length.

(**B**) Representative histology staining of colon.

(**C**) Flow cytometry analysis the percentage and number of Foxp3^+^ T_reg_ and ST2^+^Foxp3^+^ T_reg_ cells in the colon.

Data are from 2 independent experiments. Statistical comparisons were performed using two-tailed unpaired t-test. Error bars indicate mean ± SD. ns: not significant. n=5 independent biological samples. Scale bar represents 50 μm.


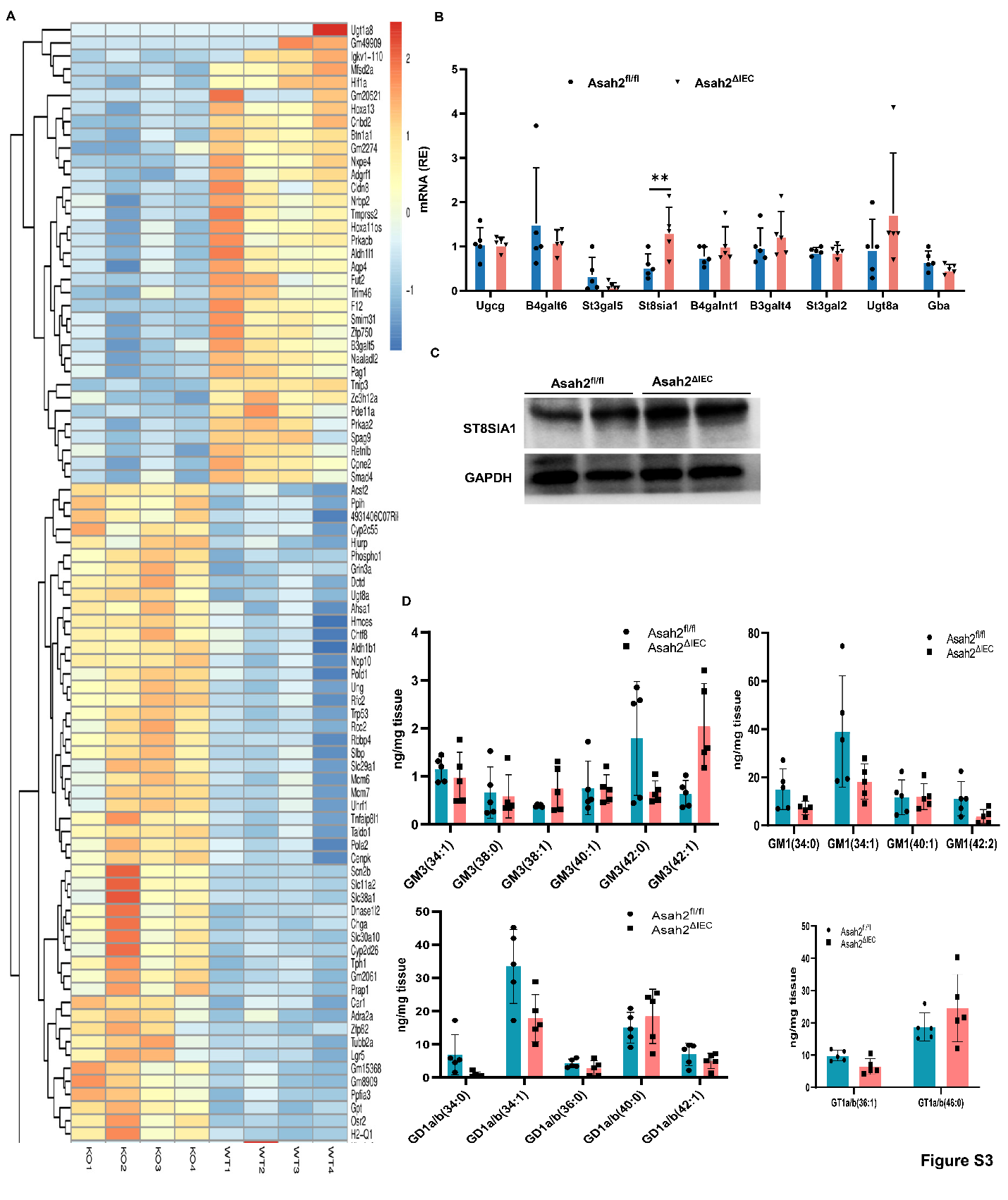


**Figure S3. Deletion of epithelial neutral ceramidase promotes GD3 production in the colon.**

(**A**) Transcriptional profiles of IECs from colon of DSS-treated *Asah2^ΔIEC^* and *Asah2^fl/fl^* mice. A heat map depicts expression levels of the major changes in genes including genes encoding ganglioside synthesis.

(**B**) Real-time PCR analysis of the genes involved in glycosphingolipid and ganglioside synthesis pathway in the colon tissues of DSS-treated *Asah2^ΔIEC^* and *Asah2^fl/fl^* mice.

(**C**) Western blot analysis of the St8sia1 expression in the IECs of colon.

(**D**) Levels of gangliosides in the colon from DSS-treated *Asah2^ΔIEC^* and *Asah2^fl/fl^* mice.

Statistical comparisons were performed using two-tailed unpaired t-test (B, D). Error bars indicate mean ± SD. **p < 0.01. n=3 (A), n=5 (B, D) independent biological samples.

**
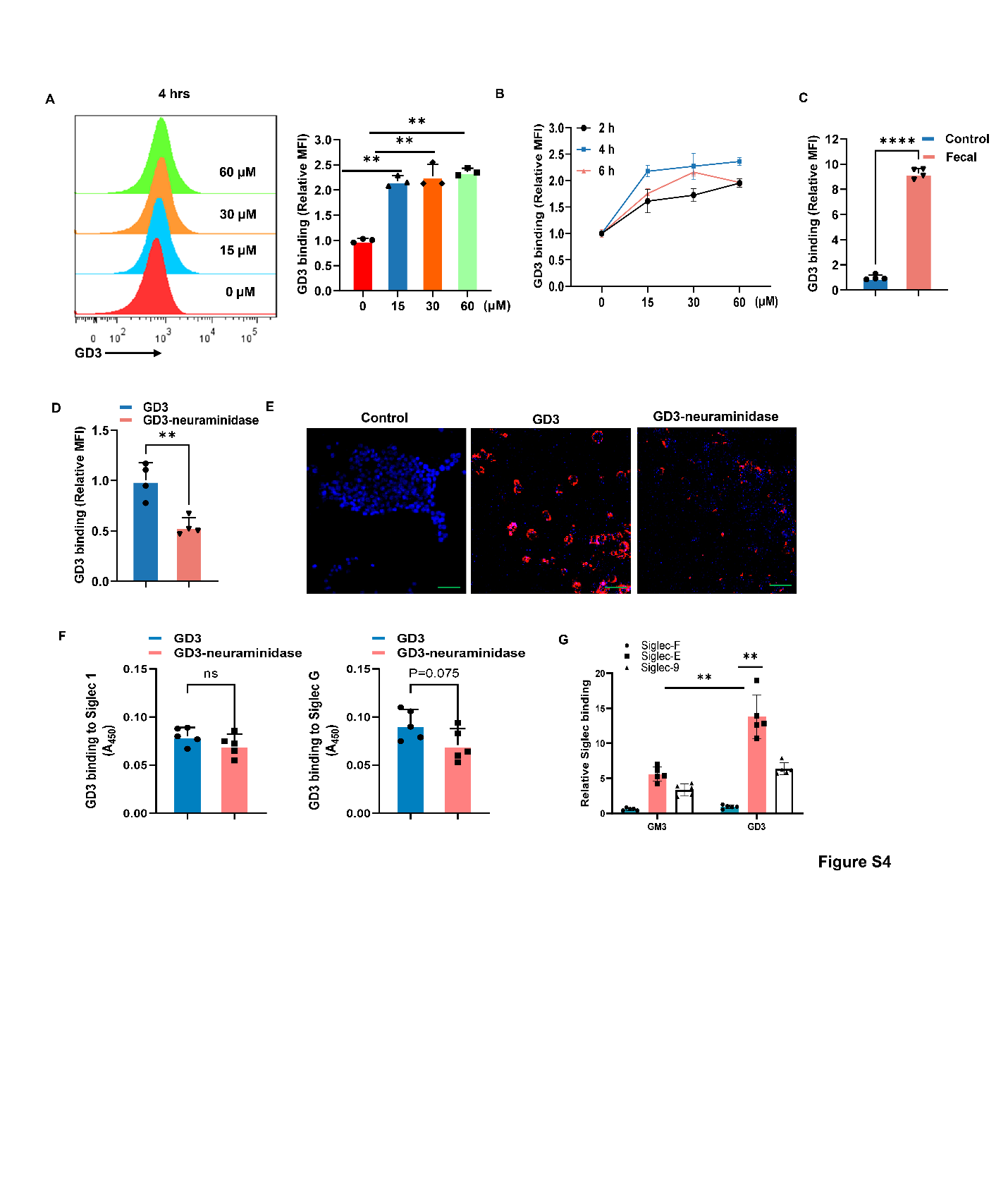
Figure S4. GD3 binds to macrophage through Siglec E.**

(**A-B**) Binding of GD3 to Raw 264.7 cells. Raw 264.7 cells were incubated with different concentrations of biotinylated GD3 for 4 hours (A) or 2-4 hours (B), then analyzed by flow cytometry; results are presented as the fold changes of mean fluorescence intensity (MFI).

(**C-D**) Flow cytometry analyzing the binding of GD3 to Raw 264.7 cells treated with fecal contents from mice with colitis (C); or the binding of GD3 and neuraminidase-treated GD3 to Raw 264.7 cells (D). Results are presented as the fold changes in mean fluorescence intensity (MFI).

(**E**) Fluorescence microscopy of Raw264.7 cells incubated with control, biotinylated GD3, neuraminidase-treated biotinylated GD3, and then stained with streptavidin-phycoerythrin. Original magnification, ×200. Scale bar represents 50 μm.

(**F**) Binding of GD3 or neuraminidase-treated GD3 to recombinant Siglec-1 or Siglec-G protein.

(**G**) Binding of GM3 or GD3 to recombinant protein Siglecs.

Statistical comparisons were performed using two-tailed unpaired t-test (C, D); one-way ANOVA with Tukey’s multiple comparisons test (A). two-way ANOVA with Sidak’s multiple comparisons test (G). Error bars indicate mean ± SD. *p < 0.05, **p < 0.01, ***p < 0.001, ****p < 0.0001. n=3 (A), n=4 (C, D), or n=5 (F, G) independent biological samples.


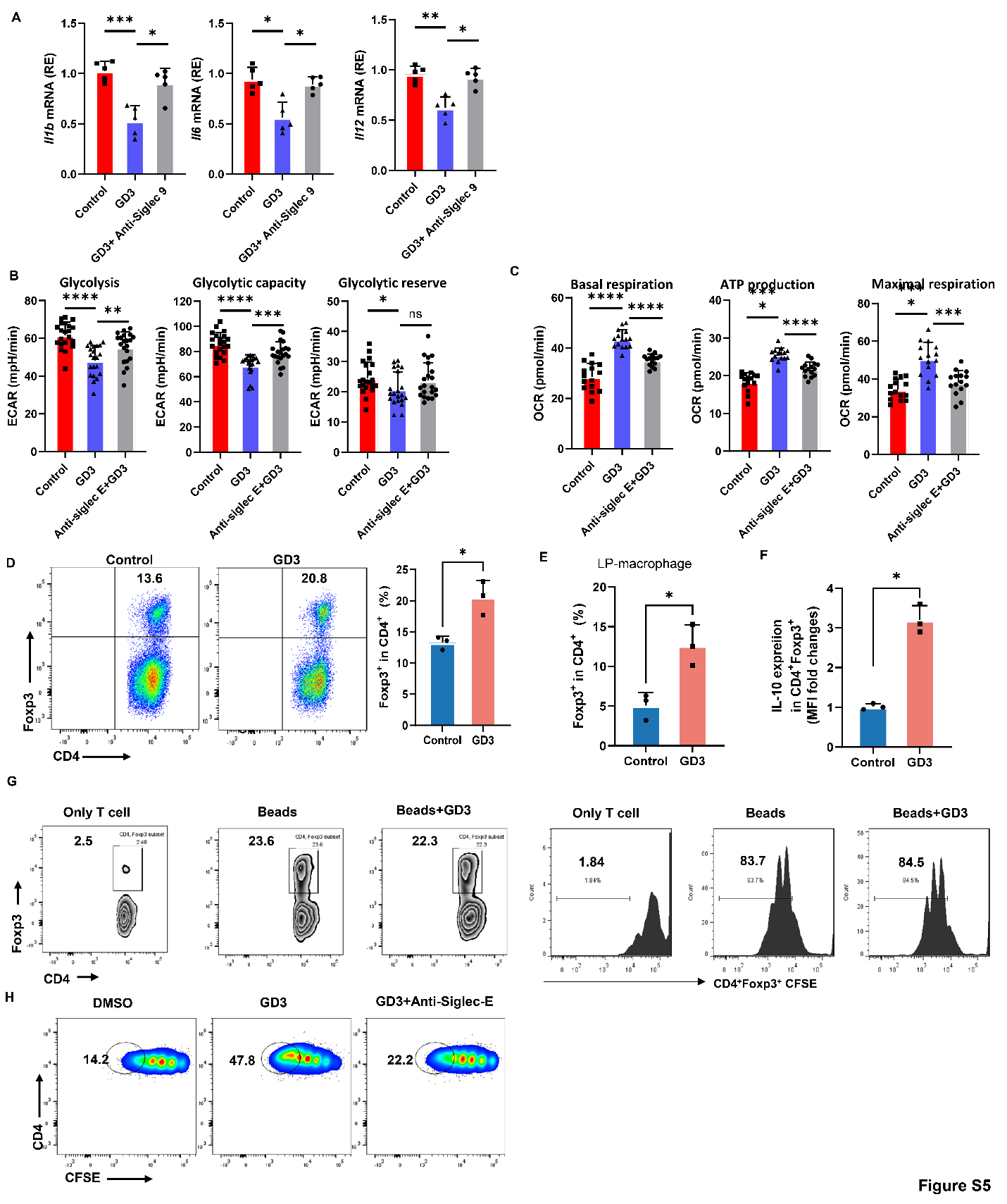


**Figure S5. GD3-Siglec E binding induces metabolic reprogramming in macrophages that promote the generation of Treg cells.**

(**A**) Real-time PCR analysis of the indicated genes in differentiated human THP-1 cells. THP-1 cells were differentiated by PMA and then treated for 3 days with a combination of cytokines including IL-4 (20ng/ml) and TGF-β (50ng/ml) in the presence or absence of 20μM GD3 with or without anti-Siglec-9 antibody.

(**B**) ECAR of glycolysis, glycolytic reserve, and capacity in BMDMs treated with fecal contents for 17 h in the presence of DMSO (Control), 20 μM GD3 or GD3+ anti-Siglec-E mAb.

(**C**) OCR of basal respiratory capacity, and maximal respiratory capacity in BMDMs treated with fecal contents for 17 h in the presence of DMSO (Control) or 20 μM GD3 or GD3+ anti-Siglec-E mAb.

(**D-E**) Frequencies of Foxp3^+^ CD4^+^ T cells after naive CD4^+^ T cells were cocultured with BMDMs (D) or macrophages from lamina propria (LP-macrophage, E) exposure to 20μM GD3 as described in **Figure 5F**.

(**F**) FACS analysis of IL-10 expression in Foxp3^+^ CD4^+^ T cells from S5E.

(**G**) Effect of GD3 on T_reg_ cell differentiation and proliferation in the absence of macrophages. Naive CD4^+^ T cells were activated with CD3/CD28 antibody-coated beads under suboptimal T_reg_ cell-inducing conditions. Cells were analyzed on day 6 by FACS.

(**H**) Assessment of cell proliferation. Naive CD4^+^ T cells were labelled with CFSE and cultured with BMDMs as in **Figure 5F**, in the presence of GD3 (20 μM). CFSE dilution in CD4 T cells was assessed on day 4 by FACS. The percentage shows the cells that underwent the indicated number of cell divisions.

Statistical comparisons were performed using two-tailed unpaired t-test (A); one-way ANOVA with Tukey’s multiple comparisons test (B, E-H). two-way ANOVA with Sidak’s multiple comparisons test (C). Error bars indicate mean ± SD. *p < 0.05, **p < 0.01, ****p < 0.0001, n=5 (A), n=3 (D-F) independent biological samples.

**
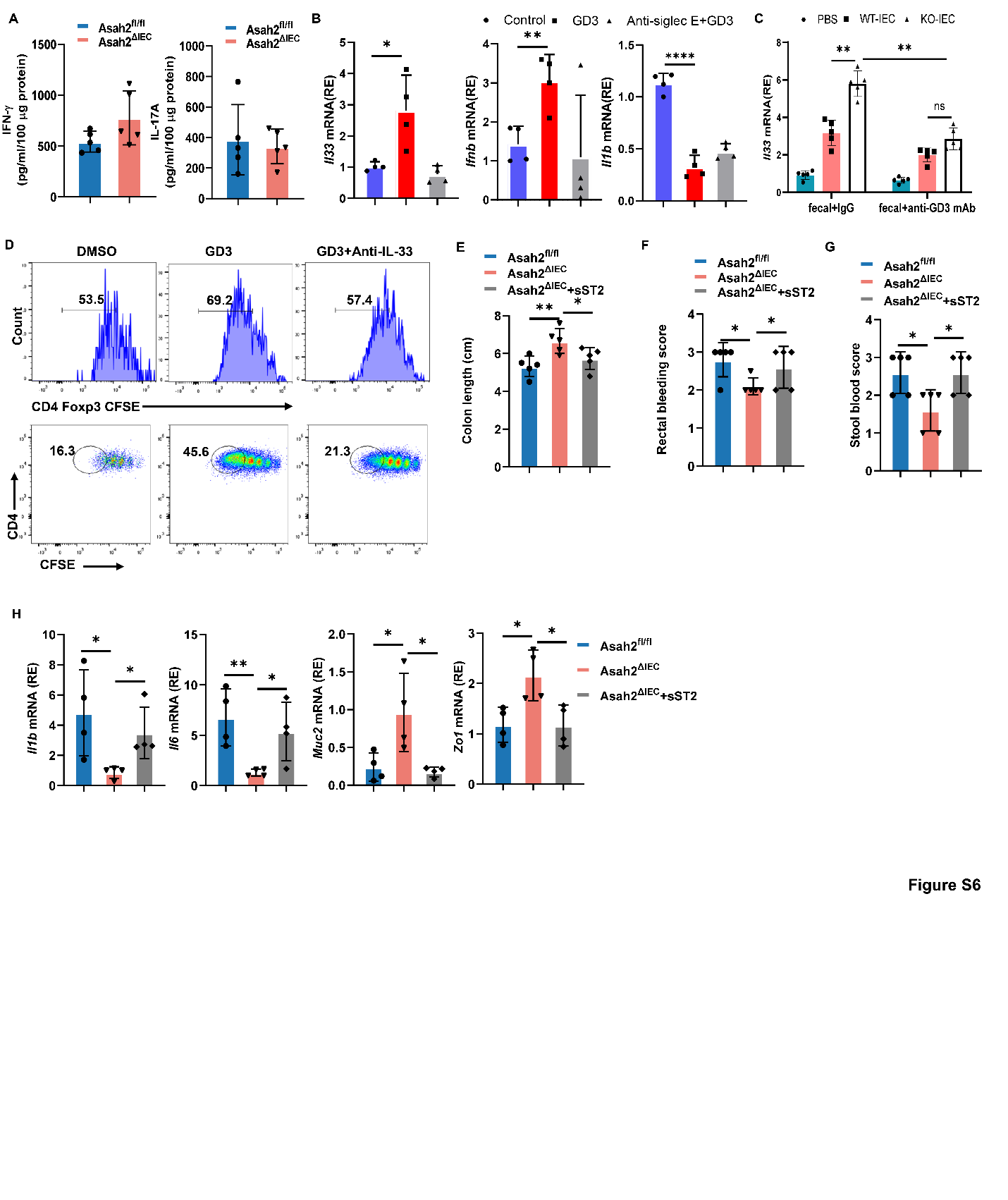
**

**Figure S6. GD3-Siglec E ligation induces macrophages to secrete IL-33 associated with colitis prevention via the induction of ST2^+^ Treg cells.**

(**A**) MSD analysis of INF-γ and IL-17A in colon tissues from DSS-treated *Asah2^ΔIEC^* and *Asah2^fl/fl^* mice (6 days post-DSS treatment).

(**B**) Real-time PCR analysis of IL-33, IFN-β, and IL-1β mRNA expression in BMDMs treated with treated with fecal contents in the presence of control vehicle, GD3 (20 μM) or GD3+ anti-Siglec E mAb.

(**C**) Real-time PCR analysis of IL-33 mRNA expression in BMDMs cocultured with colonic IECs. BMDMs were cocultured with IEC from WT (WT-IEC) and Asah2^ΔIEC^ (KO-IEC) mice with DSS-induced colitis and treated with the lysate of fecal contents for 24 hours in the presence or absence of anti-GD3 antibody.

(**D**) Assessment of cell proliferation. Naive CD4^+^ T cells were labelled with CFSE and cultured with BMDMs in the presence of GD3 (20 μM) with/without anti-IL33 mAb, as experiment described in Figure **5F**. CFSE dilution in CD4 T cells and CD4 Foxp3 T cells was assessed on day 4 by FACS. The percentage shows the cells that underwent the indicated number of cell divisions.

(**E-H**) IL-33 was blocked during the treatment of DSS via sST2. PBS or 100 μg sST2 was injected i.p. into *Asah2^ΔIEC^* on the day 4, 5, 6, 7 and 8 of the DSS challenge phase.

(**E**) Colon length.

(**F-G**) Scores for rectal bleeding (F) and blood in stools (G).

(**H**) Real-time PCR analysis of indicated genes in the colon.

Statistical comparisons were performed using two-tailed unpaired t-test (A); one-way ANOVA with Tukey’s multiple comparisons test (B, E-H). Error bars indicate mean ± SD. *p < 0.05, **p < 0.01, ****p < 0.0001. n=5 (A, C, E-G) or n=4 (B, H) independent biological samples. Scale bar represents 100 μm.


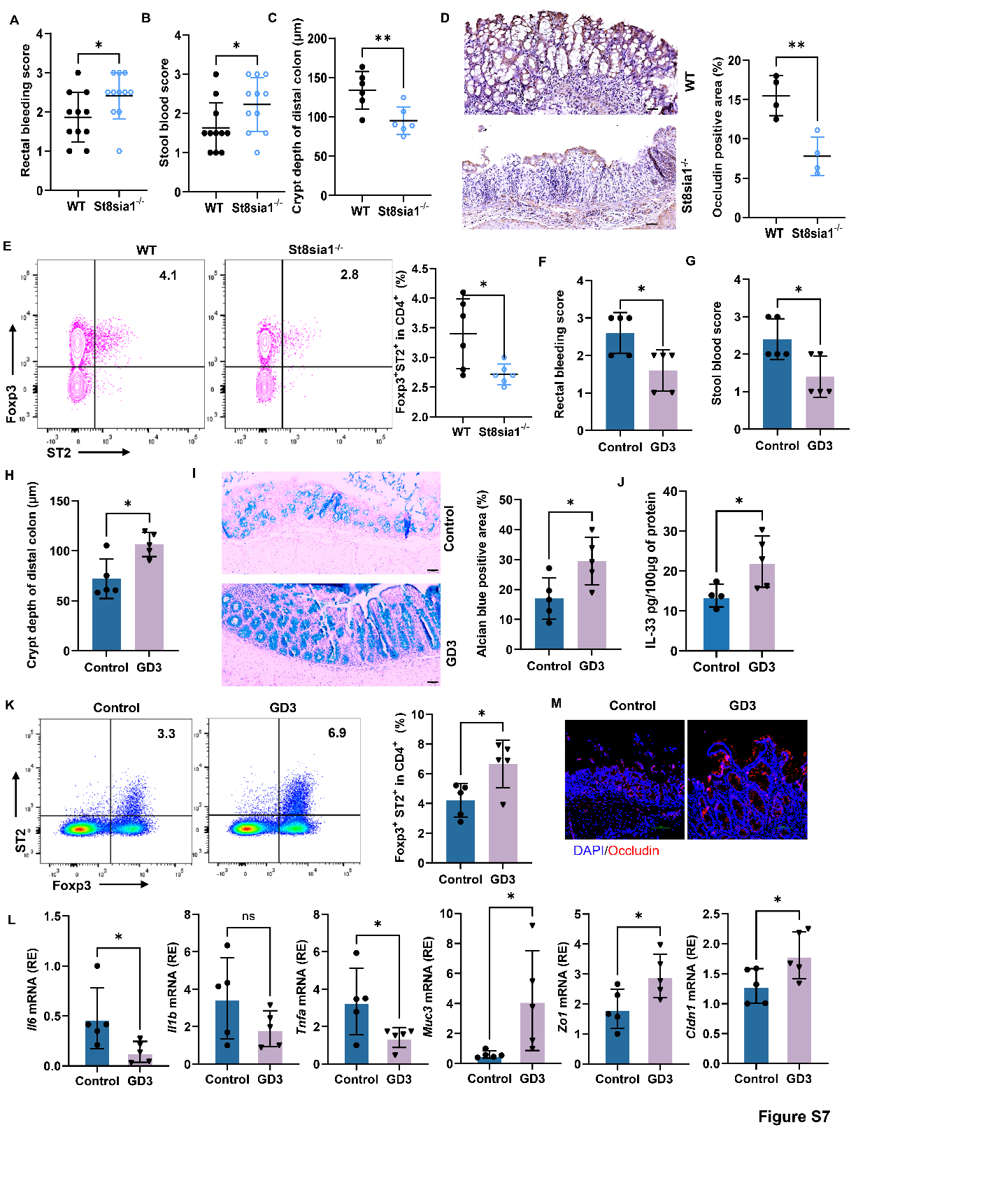


**Figure S7 St8sia1 and its metabolite GD3 control the development of colitis.**

(**A-D**) WT and *St8sia1***^-/-^** mice were treated with 2.5% DSS in drinking water for 8 days.

(**A-B**) Scores for rectal bleeding (A) and blood in stools (B).

(**C**) Crypt depth of distal colon.

(**D**) Immunohistochemistry staining of occludin protein in the colon. Scale bar represents 50 μm.

(**E**) Flow cytometry analysis of ST2^+^Foxp3^+^ T_reg_ cells.

(**F-M**) C57BL/6J mice were orally given 10 mg of extracted GD3 per day at one week before and after DSS administration.

(**F-G**) Scores for rectal bleeding (F) and blood in stools (G).

(**H**) Crypt depth of distal colon.

(**I**) Alcian blue staining of colon. Scale bar represents 50 μm.

(**J**) ELISA analysis of IL-33 in colon tissues of DSS-treated C57BL/6J mice for 8 days with/without GD3 treatment.

(**K**) Flow cytometry analysis of the percentage of ST2^+^Foxp3^+^ T_reg_ cells in the colon.

(**L**) Real-time PCR analysis of indicated genes in the colon.

(**M**) Immunofluorescence staining of occludin protein in the colon. Scale bar represents 100 μm.

Statistical comparisons were performed using two-tailed unpaired t-test; Error bars indicate mean ± SD. *p < 0.05, **p < 0.01. n=5 (E-K, L) independent biological samples. Scale bar represents 100 μm.
